# Comparative gene retention analysis in barley, wild emmer, and bread wheat pangenome lines reveals factors affecting gene retention following gene duplication

**DOI:** 10.1186/s12915-022-01503-z

**Published:** 2023-02-06

**Authors:** Yong Jia, Mingrui Xu, Haifei Hu, Brett Chapman, Calum Watt, B. Buerte, Ning Han, Muyuan Zhu, Hongwu Bian, Chengdao Li, Zhanghui Zeng

**Affiliations:** 1 Western Crop Genetic Alliance, College of Science, Health, Engineering and Education, Murdoch University, 90 South Street, Murdoch, WA 6150 Australia; 2 Western Australian State Agricultural Biotechnology Centre, Murdoch University, 90 South Street, Murdoch, WA 6150 Australia; 3 College of Life and Environmental Sciences, Hangzhou Normal University, Hangzhou, 311121 China; 4 Intergrain Pty Ltd, Bibra Lake, WA 6163 Australia; 5 Institute of Genetic and Regenerative Biology, Key Laboratory for Cell and Gene Engineering of Zhejiang Province, College of Life Sciences, Zhejiang University, Hangzhou, 310058 China; 6 Department of Primary Industries and Regional Development, 3-Baron-Hay Court, South Perth, WA 6151 Australia; 7 Zhejiang Provincial Key Laboratory for Genetic Improvement and Quality Control of Medicinal Plants, Hangzhou, 311121 China

**Keywords:** Gene duplication, Gene retention, Homogentisate phytyltransferase, Pangenome, Plant defense responses, Positive selection, Relaxed selection, Vitamin E

## Abstract

**Background:**

Gene duplication is a prevalent phenomenon and a major driving force underlying genome evolution. The process leading to the fixation of gene duplicates following duplication is critical to understand how genome evolves but remains fragmentally understood. Most previous studies on gene retention are based on gene duplicate analyses in single reference genome. No population-based comparative gene retention analysis has been performed to date.

**Results:**

Taking advantage of recently published genomic data in *Triticeae*, we dissected a divergent *homogentisate phytyltransferase* (*HPT2*) lineage caught in the middle stage of gene fixation following duplication. The presence/absence of *HPT2* in barley (diploid), wild emmer (tetraploid), and bread wheat (hexaploid) pangenome lines appears to be associated with gene dosage constraint and environmental adaption. Based on these observations, we adopted a phylogeny-based orthology inference approach and performed comparative gene retention analyses across barley, wild emmer, and bread wheat. This led to the identification of 326 *HPT2-pattern-like* genes at whole genome scale, representing a pool of gene duplicates in the middle stage of gene fixation. Majority of these *HPT2-pattern-like* genes were identified as small-scale duplicates, such as dispersed, tandem, and proximal duplications. Natural selection analyses showed that *HPT2-pattern-like* genes have experienced relaxed selection pressure, which is generally accompanied with partial positive selection and transcriptional divergence. Functional enrichment analyses showed that *HPT2-pattern-like* genes are over-represented with molecular-binding and defense response functions, supporting the potential role of environmental adaption during gene retention. We also observed that gene duplicates from larger gene family are more likely to be lost, implying a gene dosage constraint effect. Further comparative gene retention analysis in barley and bread wheat pangenome lines revealed combined effects of species-specific selection and gene dosage constraint.

**Conclusions:**

Comparative gene retention analyses at the population level support gene dosage constraint, environmental adaption, and species-specific selection as three factors that may affect gene retention following gene duplication. Our findings shed light on the evolutionary process leading to the retention of newly formed gene duplicates and will greatly improve our understanding on genome evolution via duplication.

**Supplementary Information:**

The online version contains supplementary material available at 10.1186/s12915-022-01503-z.

## Background

Gene and gene evolution are of paramount importance to understand how life has evolved and functions on earth. The major mechanism of gene evolution is through gene duplication [1–3], which is a common evolutionary process in the genomes of all living organisms [4, 5]. Gene duplication provides raw genetic materials that mutation and selection can act on and plays a critical role in genome evolution [6], phenotypic diversification [7, 8], and environmental adaptation [9]. It has been reported that 38% of the annotated genes in human genome [10], 41% in fruit fly [11], 65% in *Arabidopsis* [12], 30% in yeast [11], and 17% in bacteria [11] are gene duplicates. Comparative genomics analyses across different life forms have revealed that gene families are generally conserved [11, 13], leading to the suggestion that gene duplication, instead of gene invention, is the major driving force underlying genome evolution [6, 14].

Following a duplication event, most of the newly formed gene duplicates experience relaxed selection constraints and are rapidly eliminated via gene pseudogenization or gene deletion [15–17]. The classical population genetic theory has predicted that only a small number of new duplicates can be retained and fixed in the population [17–19], mainly due to the genetic drift effect. On the other hand, several studies have suggested that gene duplication events may occur as frequently as common genetic variations, such as single-nucleotide polymorphism (SNP) [17, 20–22]. These studies suggest that gene duplication, gene loss, and gene retention occur concurrently in a dynamic manner. Given the extremely low chance of a single duplication event becoming fixed in a population [16, 19], it raises the question: what type of gene can escape the gene loss fate and be retained? Or what impacting factors may determine the gene retention process?

To date, this question has only been partially answered. By analyzing duplicated gene pairs formed by ancient polyploidy events, pioneering studies in *Arabidopsis* [23, 24] showed that genes retained in duplicate are not distributed evenly among Gene Ontology (GO) or gene functional categories. Generally, they observed that genes encoding transcription factors and signal transduction proteins tend to be preferentially retained. This observation was corroborated by later findings in other organisms including rice, poplar, tetraodon, and yeast [25–27]. In addition, it has also been noticed that the gene retention process varies for duplicates resulted from whole genome duplications (WGDs) and small-scale duplications (SSDs, such as tandem duplication) [28, 29] and also seems to be affected by various other factors such as protein properties, transcription level, and epigenetics [29–32]. Along with these findings, various models and hypotheses have been proposed to explain the biased process of gene retention following duplication [5, 33]. Most recently, a new “Structural and functional entanglement” theory was proposed to explain how gene duplicate can be retained in yeast [5, 34]. This highlights the complex nature of gene evolution via duplication, which remains fragmentally understood.

One notable limitation for these earlier studies [23–26, 31, 32, 35, 36] is that they were based on gene duplicate analyses in single or a couple reference genomes. Gene duplicates present in individual reference genome are mainly caused by ancient ploidy or other long-term duplication events [23]. Therefore, the observed gene retention pattern may not reflect directly how gene duplicates can be retained. In addition, it is also unclear how much role that natural selection has played during the gene retention process. Therefore, we ask the question: is it possible to analyze how gene duplicates are retained in a real-time manner? Since the fixation process of gene duplicates occurs dynamically in the context of a large population, we reason that analyzing the gene retention pattern in the pangenomes would answer this intriguing question. Luckily, the recent revelation of pangenome data in more and more species provides a valuable opportunity that is needed.

Compared to other organisms, plants are particularly prone to polyploidization, thus displaying a higher rate of gene duplication [37, 38], thereby providing a more suitable model to study how gene evolves via duplication. In this study, we aim to characterize the gene evolution process via duplication, with a particular focus on how gene duplicates are retained in the population context. We take advantage of recently published genomic data in barley [39], wild emmer [40, 41], and bread wheat [42], to dissect a convincing example of gene caught in the middle stage of gene fixation following duplication. Next, we expanded the search to the whole genome level and identified a set of candidate genes that have been retained in a biased manner across species. Critical gene functional and natural selection pressure profiles for these preferentially retained gene duplicates were obtained. We introduce a population-based gene retention analysis, as a novel genomic approach to take a snapshot of the gene evolution process via duplication. Our analyses provide novel insights into the long-debated process of gene retention after duplication.

## Results

### Identification of a divergent lineage of homogentisate phytyltransferase for vitamin E biosynthesis in Triticeae and Aveninae

*Homogentisate phytyltransferase* (*HPT*) is a critical gene responsible for tocopherol production in the vitamin E biosynthetic pathway (Fig. S1). We previously observed that *HvHPT* knock-out mutation cannot eliminate tocopherol production [43], suggesting multiple copies of *HPT* may be present in barley genome. Thus, we performed a genome-wide screening of UbiA prenyltransferase-encoding gene (PF01040) in 22 *Poales* species, including 8 core *Pooideae* (*Hordeum vulgare*, *Triticum aestivum*, *Triticum dicoccoides*, *Triticum urartu*, *Thinopyrum intermedium*, *Aegilops tauschii*, *Secale cereal*, *Avena eriantha*), 5 *Brachypodieae* (*Brachypodium distachyon*, *Brachypodium hybridum*, *Brachypodium mexicanum*, *Brachypodium stacei*, *Brachypodium sylvaticum*), 4 *Panicoideae* (*Panicum hallii*, *Sorghum bicolor*, *Setaria italic*, *Zea mays*), *Oryza sativa*, and *Ananas comosus*. Three additional *Pooideae* species *Nardus stricta*, *Stipa lagascae*, and *Melica nutans* were also searched to resolve *HPT* evolution. A total of 62 *HPT* homologous genes were identified (Additional file 1: Table S1).

**Fig. 1.**
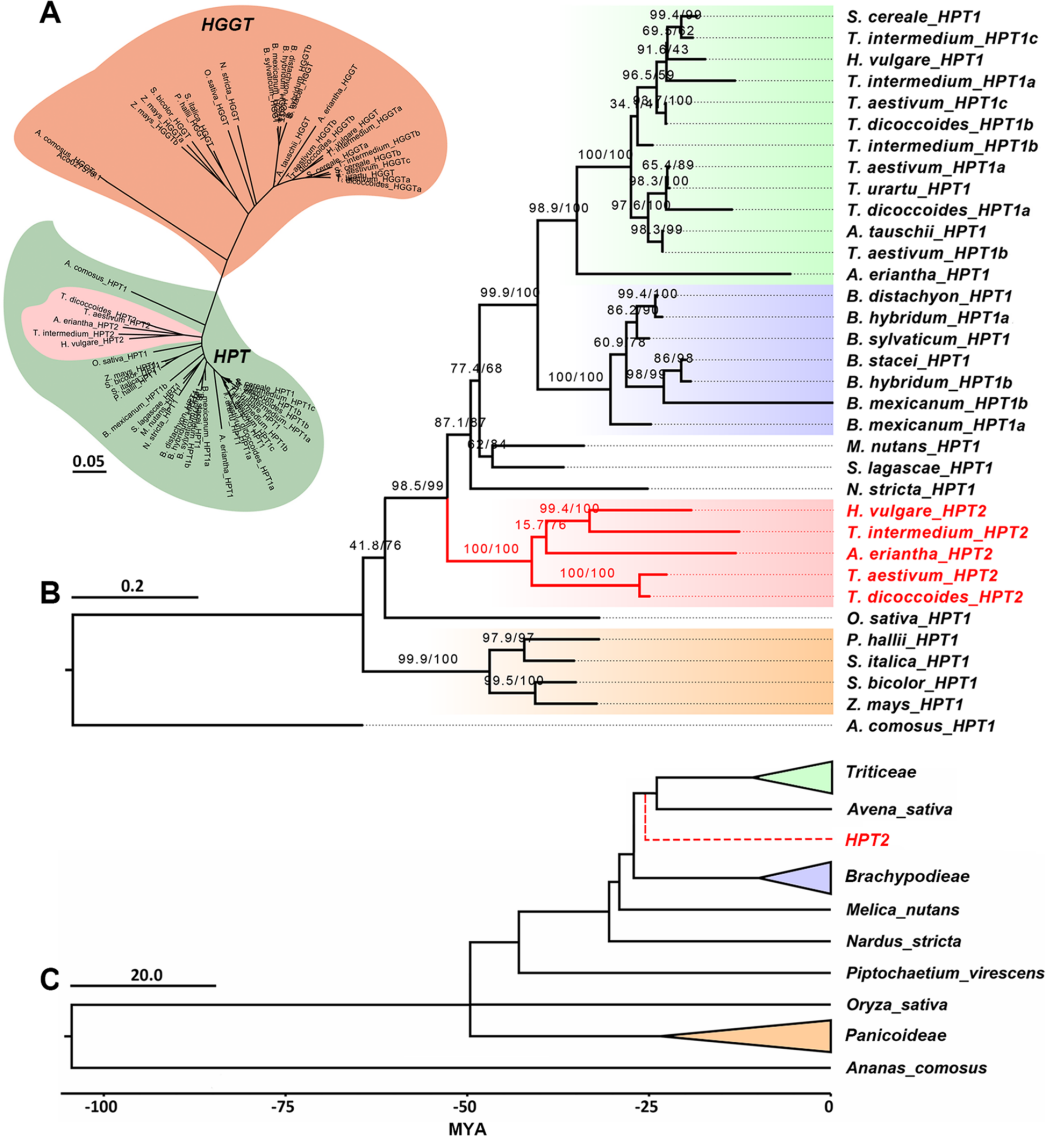
Phylogeny analyses of *HPT* homologous genes in *Poales* species. **A** Neighbor joining phylogeny displaying the divergence between *HPT* (light green) and *HGGT* (brown) in 22 *Poales* species. The presence of a divergent *HPT* lineage (*HPT2*) was highlighted in light red. **B** Maximum likelihood phylogeny for the HPT-encoding genes in 22 *Poales* species. One thousand times bootstrapping and SH-like approximate likelihood ratio test supports were labelled above each branch. The *HPT2* lineage were colored in red. **C** Calibrated species phylogeny for the *Poales* species. The deduced emergence time of *HPT2* was indicated in red dashed line (see Additional file 2 for detailed sequence alignment and tree files)

A preliminary neighbor joining (NJ) phylogeny was developed to differentiate *HPT* from its close homolog homogentisate geranylgeranyl transferase (*HGGT*) (responsible for tocotrienol production, Additional file 1: Fig. S1). The target genes grouped into 2 major clusters, corresponding to *HPT* and *HGGT*, respectively (Fig. 1A). A more robust maximum likelihood (ML) phylogeny (Fig. 1B) was constructed for *HPTs* specifically. The overall topology was consistent with the species phylogeny (Fig. 1C). Consistent with our speculation, *Pooideae* species *HPTs* divided into two major subclades. The first *HPT* subclade (named as *HPT1*) covers all target species in this study and contains the previously characterized *HPTs* [43–45]. The second *HPT* subclade (named as *HPT2*, Fig. 1B) only covers 5 species from *Triticeae* (barley, bread wheat, wild emmer, *T. intermedium*) and *Aveninae* (*A. eriantha*) and represents a novel observation. The presence of *HPT2* in *Aveninae* and *Triticeae* species indicated that *HPT2* emerged in their common ancestor. None of the five *Brachypoideae* species contain *HPT2* (Fig. 1B), supporting the emergence of *HPT2* after the split of *Brachypodieae*, corresponding to ~ 25 million years ago (Fig. 1C). In the ML tree, *HPT2* diverges earlier than *N. stricta*, *S. lagascae*, *M. nutans*, and *Bracypoideae HPT1s*, suggesting that *HPT2s* have very divergent sequences from *HPT1s*. Indeed, protein sequence identity analysis showed that HPT2s only have an average 78% identity with *Pooideae* HPT1s, lower than the 81% identity between *Panicoideae* HPT1s and *Pooideae* HPT1s (Additional file 1: Table S2).

### *HPT2* resulted from dispersed gene duplication and is partially retained in the pangenomes of barley, wild emmer, and bread wheat

**Fig. 2.**
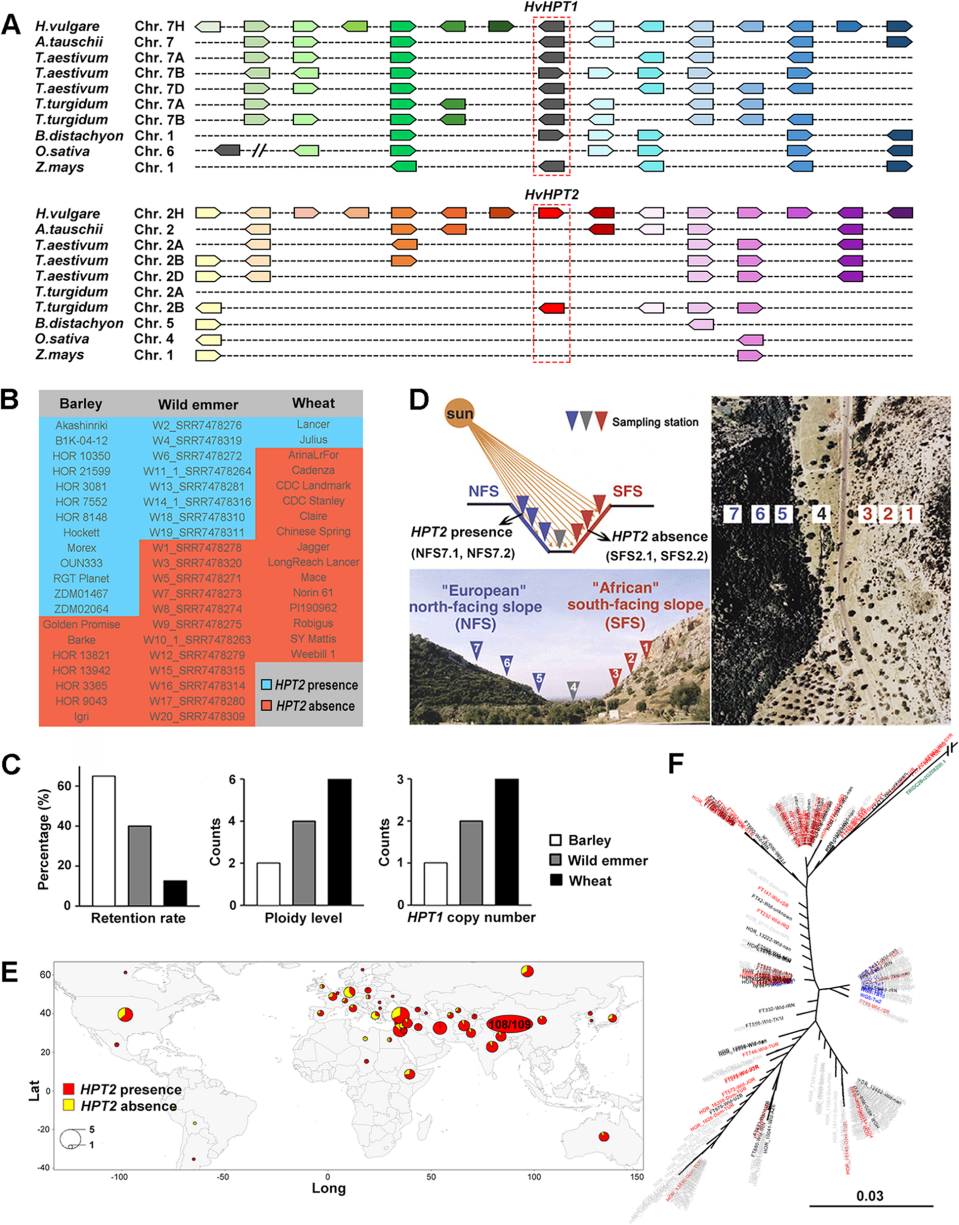
Synteny, partial retention, and evolutionary analyses of *HPT2*. **A** The synteny of *HPT1* and *HPT2* in common *Pales* species using barley genome as references (see Additional file 3 for gene ids). **B** Presence of and absence of *HPT2* in barley pangenome (20 lines), representative wild emmer (20 lines), bread wheat pangenome (16 lines). **C** Bar charts displaying the negative correlation of *HPT2* retention rates with the ploidy level and *HPT1* copy number in barley, wild emmer, and bread wheat. **D** The presence and absence of *HPT2* in wild barley lines collected from the north-facing slope (NFS) and south-facing slope (SFS) of the Evolution Canyon in Israel (Fig. reproduced from reference [46]). **E** Geographical map displaying the presence (red) and absence (yellow) of *HPT2* in the 300 barley WGS lines and 109 Tibetan barley lines (distinguished in oval). **F** ML phylogeny of *HvHPT2s* in barley pangenome lines based on SNP variations (Additional file 4). *HPT2* from wild emmer (TRIDC2Bv2G208200, highlighted in green) was used as outgroup to resolve the evolutionary origin of *HvHPT2* in barley. *HvHPT2s* in barley lines near the Fertile Crescent in Israel (red; including Israel, Jordan, Lebanon, Syria, Turkey, and Iraq), Tibetan barley lines (blue), other wild barley (black) and domesticated barley (gray) were distinguished

Synteny analyses in 7 *Poales* reference genomes (Fig. 2A) showed that *HPT1* is generally conserved in a collinear block, with the exception of *OsHPT1* which is located in a different genetic region from *HPT1s* in other species. In contrast, *HPT2* is only preserved in reference genomes of barley and wild emmer and displays a clear gene insertion pattern. In barley, *HvHPT1* and *HvHPT2* were found on two different chromosomes with no synteny with each other. These observations support *HPT2* as a dispersed duplication from *HPT1*.

During gene screening, we noticed that *HPT2* is absent in some barley germplasm lines. To obtain a complete presence/absence profile, we searched *HPT2* in the pangenome lines of barley, wild emmer, and bread wheat. Results (Fig. 2B) showed that *HPT2* is conserved in 13 out of the 20 barley pangenomes (65%), 8 out of the 20 representative wild emmer lines (40%), and 2 out of the 16 wheat pangenomes (12.5%). In particular, the presence of *HPT2* only in wheat cultivars Lancer (*TraesLAC2B03G00984720.1*) and Julius (*TraesJUL2B03G01032940.1*) was validated by tblastn search against the wheat pangenome assemblies. Interestingly, the retention rates of *HPT2* are negatively correlated (coefficient = −0.9996) with *HPT1* copy numbers in barley (1), wild emmer (2), and bread wheat (3) (Fig. 2C), suggesting that the retention of *HPT2* may be affected by a varied *HPT1* gene dosage constraint. Due to the absence of *HPT2* in bread wheat reference genome Chinese Spring, we designed a gene-specific marker for *HPT2* in bread wheat and genotyped a collection of 113 bread wheat accessions (mainly Australian cultivars). Results (Additional file 1: Table S3) showed that 12 out of the 113 (10.6%) accessions contain the *HPT2* gene, very close to the observation in the 16 wheat pangenome lines.

The Evolution Canyon (EC) in Israel is comprised of two abutting slopes (Fig. 2D, distanced by 250m) with contrasting micro-environments: north002Dfacing slope (NFS, cool and humid) and south-facing slope (SFS, hot and dry), and has been used as a model to study the environmental adaption of various organisms [46–49]. During *HPT2* gene scanning, we observed that *HPT2* is only present in wild barley lines from NFS but absent in SFS (Fig. 2D). This observation suggests that the retention of *HPT2* may be related to environmental adaption. In plants, vitamin E synthesis plays a major role in cold tolerance and low-temperature response [50]. To investigate whether *HPT2* may be preferentially retained in plants grown under cold environment, we scanned 109 wild barley lines from Tibet (low temperature and high UV). Results (Fig. 2E) showed that *HvHPT2* is present in 108 out of the 109 Tibetan barley lines (99.1%), supporting *HPT2*’s role in environmental adaptation. Furthermore, a geographic distribution of the presence-absence of *HvHPT2* in 300 barley resequencing lines was generated (Fig. 2E), which showed that the retention of *HPT2* (237 of the 300 lines, ~ 79%) is geographically imbalanced, implying a potential local environmental selection pattern.

To deduce the origin of *HvHPT2* in barley, a ML tree was constructed based on *HvHPT2* SNPs. *HPT2* (*TRIDC2Bv2G208200*) from wild emmer was used as outgroup. Results (Fig. 2F) showed that *HvHPT2* in wild barley lines from Turkey (2), Syria (1), and Iraq (1) diverged first, suggesting that these wild barley lines may contain the earliest *HvHPT2* haplotypes. In contrast, the 10 Tibetan wild barley lines formed a distant cluster, suggesting that the *HvHPT2* haplotype in Tibetan barley may have evolved at a later stage.

### *HPT2* underwent an interplay of purifying, relaxed, and positive selection

The ratio (*ω*) of non-synonymous (*Ka*) to synonymous (*Ks*) substitutions is used to assess gene selection pressure, whereby *ω* < 1, *ω* = 1, and *ω* > 1 indicate purifying, neutral, and positive selections, respectively. Four phylogeny lineages: *HPT2s, Triticeae-Aveninae HPT1s*, *Brachypodieae HPT1s*, and *Panicoideae HPT1s* (highlighted in Fig. 1B) were specified for *ω* calculation, corresponding to *ω*_*HPT2*_, *ω*_*TA*_, *ω*_*BR*_, and *ω*_*PC*_, respectively.

Under the branch-specific method (Table 1), likelihood ratio tests (LRTs) showed that the two-ratio model (*ω*_*HPT2*_≠*ω*_*BR*_=*ω*_*TA*_=*ω*_*PC*_) fitted the data significantly better (*p* = 0.0014 with 2*∆l* = 10.22 and df = 1) than the one-ratio model (*ω*_*HPT2*_=*ω*_*BR*_=*ω*_*TA*_=*ω*_*PC*_). In contrast, the three-ratio model (*ω*_*HPT2*_≠*ω*_*BR*_=*ω*_*TA*_≠*ω*_*PC*_) and the four-ratio model (*ω*_*HPT2*_≠*ω*_*BR*_≠*ω*_*TA*_≠*ω*_*PC*_) were not better than the two-ratio model (*p* =0.2059 with 2*∆l* = 1.6 and df = 1, *p* = 0.6005 with 2*∆l* = 1.02 and df = 2, respectively). Thus, the two-ratio model (*ω*_*HPT2*_≠*ω*_*BR*_=*ω*_*TA*_=*ω*_*PC*_) was identified as the besting fitting model, which indicates that *HPT2* was under different selection pressure (*ω*_*HPT2*_=0.81568) from *HPT1* lineages (*ω*_*BR*_=*ω*_*TA*_=*ω*_*PC*_=0.28457). *ω*_*BR*_=*ω*_*TA*_=*ω*_*PC*_=0.28457 showed that the *HPT1* lineages were under strong purifying selection pressure, consistent with their strict conservation in all target species. In contrast, *ω*_*HPT2*_=0.81568 indicates *HPT2s* has clearly relaxed selection pressure than *HPT1s*.

**Table 1:** Natural selection tests on *HPT* genes

Model	np	***l*** = ln L	Estimates of parameters	Positively selected sites
**One-ratio**				
*ω*_*HPT2*_ = *ω*_*BR*_ = *ω*_*TA*_ = *ω*_*PC*_	1	−8204.98	*ω*_*HPT2*_=*ω*_*BR*_=*ω*_*TA*_=*ω*_*PC*_ = 0.29798	Not allowed (NA)
**Branch-specific models**				
*ω*_*HPT2*_ ≠ *ω*_*BR*_ = *ω*_*TA*_ = *ω*_*PC*_ (2 ratio)	2	−8199.87	*ω*_*HPT2*_=0.81568; *ω*_*BR*_=*ω*_*TA*_=*ω*_*PC*_=0.28457	NA
*ω*_*HPT2*_ ≠ *ω*_*BR*_ = *ω*_*TA*_ ≠ *ω*_*PC*_ (3 ratio)	3	−8199.06	*ω*_*HPT2*_=0.75587; *ω*_*PC*_=0.28095 *ω*_*HPT2*_=*ω*_*TA*_= 0.48924	NA
*ω*_*HPT2*_ ≠ *ω*_*BR*_ ≠ *ω*_*TA*_ ≠ *ω*_*PC*_ (4 ratios)	4	−8199.36	*ω*_*HPT2*_= 0.81975; *ω*_*BR*_= 0.24071; *ω*_*TA*_= 0.18172; *ω*_*PC*_= 0.28853	NA
**Branch-site-specific models** (selection test of *HPT2* as the foreground lineage)				
Model A (4 site classes)	4	−8034.75	*p*_0_=0.65921, *p*_1_=0.25827 (*p*_2_+*p*_3_=0.08252); *ω*_0_=0.09024, (*ω*_1_=1.0), *ω*_2_=4.92076	Selected sites in HPT2: 9P, 79M, 89N, 100T, 103D, 149S, 164S, 167D, 204F, 212L, 217I, 246V, 270M, 271A, 356V (Probability > 0.6)
Model A Null (4 site classes)	3	−8036.70	*p*_0_=0.55094, *p*_1_=0.21917 (*p*_2_+*p*_3_=0.22990), *ω*_0_=0.08916 (*ω*_1_=1.0, *ω*_2_=1.0)	NA

In one-ratio and branch-specific models, *ω*_*HPT2*_, *ω*_*BR*_, *ω*_*TA*_, and *ω*_*PC*_ stand for *Ka*/*Ks* values for *HPT2*, *Brachipoideae HPT1*, *Triticeae-Aveninae HPT1*, and *Panicoideae HPT1* branches in Fig. 1B. In the site-specific model M1, two site classes were specified: highly conserved sites (*ω*_0_) and neutral sites (*ω*_1_=1). For the branch-site models, *HPT2* was specified as the foreground group. In the branch-site model A, four site classes were specified. The first two classes have *ω* ratios of *ω*_0_ and *ω*_1_ respectively, corresponding to highly conserved sites and neutral sites across all lineages. In the other two site classes, the background lineages have *ω*_0_ or *ω*_1_ while the foreground lineages have *ω*_2_. *p*_0_, *p*_1_, and *p*_2_ represent the percentages of the corresponding site classes. Np: number of parameters. L: likelihood value. Amino acid sites were numbered according to HvHPT2 (HORVU.MOREX.r2.2HG0173050.1) in barley. (see Additional file 5 for detailed data)

To test if *HPT2* has been affected by positive selection, we applied the branch-site models, which allow *ω* to vary across both branches and amino acid sites. Results (Table 1, Model A) showed that 15 amino acid sites (9P, 79M, 89N, 100T, 103D, 149S, 164S, 167D, 204F, 212L, 217I, 246V, 270M, 271A, 356V; numbered according to HvHPT2) were found to be under positive selection (*ω*_2_=4.92076). LRTs showed that Model A fitted the data significantly better (*p* = 0.0486 with 2*∆l* = 3.89 and df = 1) than its null hypothesis (Table 1, Model A Null, *ω*_2_=1), suggesting these amino acid sites were indeed under positive selection. In contrast, when the *Triticeae-Aveninae HPT1s* were tested for positive selection, no amino acid site could be identified under positive selection.

### *HvHPT2* shares functional redundancy with *HvHPT1* in vitamin E biosynthesis

**Fig. 3.**
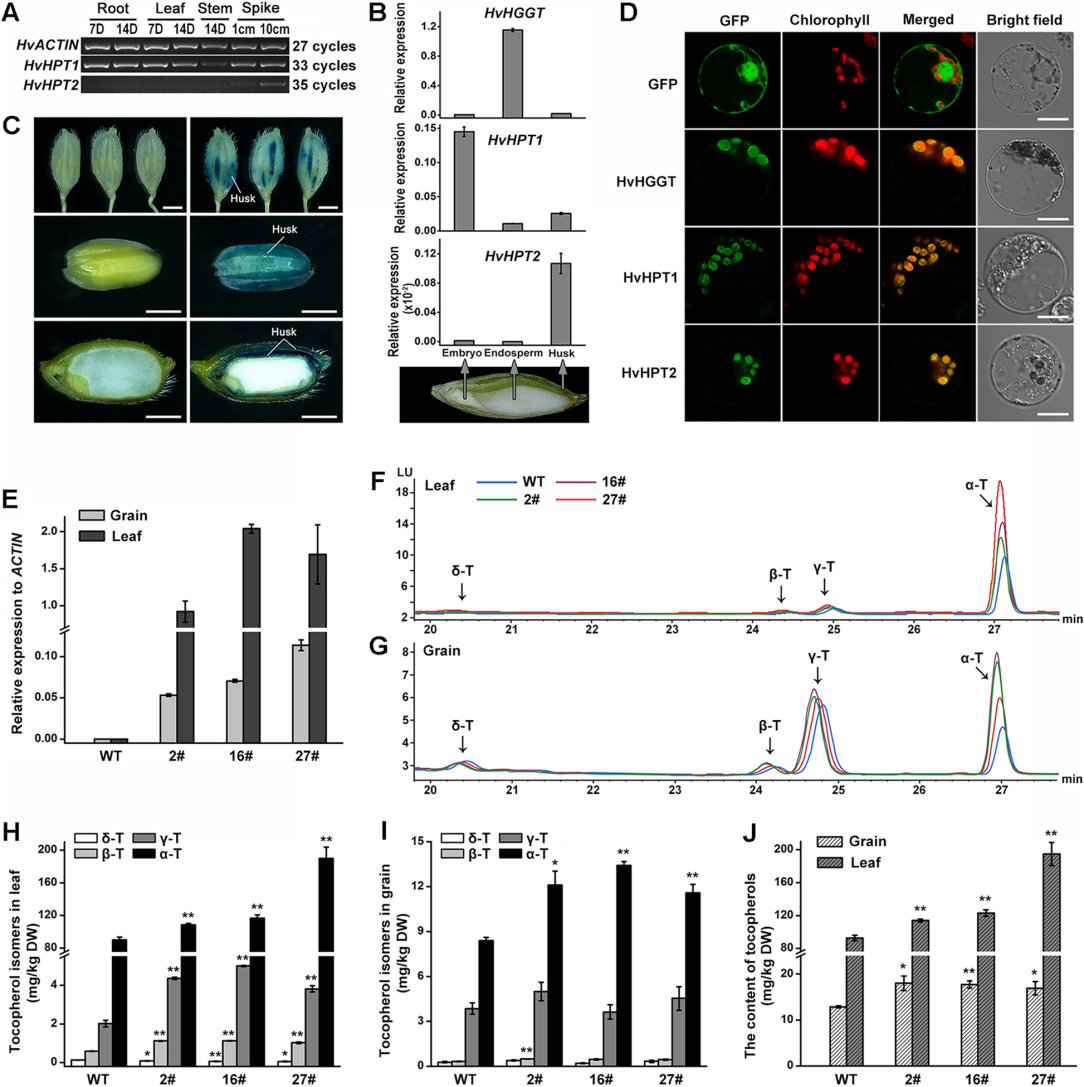
Gene expression pattern, subcellular localization of *HvHPT2*, and tocopherol levels in *HvHPT2* overexpression transgenic lines. **A** Semi-quantitative RT-PCR of *HvHPT1* and *HvHPT2* in different tissues of barley. **B** Quantitative RT-PCR was applied to detect the expression level of *HvHGGT*, *HvHPT1*, and *HvHPT2* in embryo, endosperm, and husk of barley grain (16 days after pollination, DAP), respectively. **C***β*-Glucuronidase (GUS) reporter gene expression in transgenic rice plants expressing the *HvHPT2* promoter-GUS constructs. The spike of heading stage and the grains of 14 DAP were excised and stained with GUS solution. Bar: 2mm. **D** Transient expression of proteins with GFP-tag in protoplast cells of barely. GFP fluorescence, chlorophyll autofluorescence, merged signals, and bright field are indicated above the panels. Bar: 10 μm. **E** The expression level of *HvHPT2* in transgenic barley leaves and grains. **F, G** HPLC profiles of tocopherol isomers in the leaf and grain of wild-type barley and its transgenic lines (*35S:HPT2*_2#, 16# and 27#), respectively. **H** The content of four tocopherol isomers in leaf. **I** The content of four tocopherol isomers in grain. **J** The total content of tocopherols in leaf and grain. The leaves of 2-month-old T_1_ transgenic lines and their mature grains were used for HPLC analysis. Fifty-milligrams of leaf or grain powder was used in each replicate. The means and standard errors of at least three replicates are presented. Asterisks (* or **) indicate a significant difference between the wild-type and transgenic lines at *P* < 0.05 or *P* < 0.01, respectively, as determined by Student’s *t* tests. D, day; DW, dry weight; T, tocopherol

To verify gene dosage constraint hypothesis on *HPT2* retention, we investigated if *HPT2* shares functional redundancy with *HPT1*. At the transcriptional level, semi-RT-PCR (Fig. 3A) showed that *HvHPT2* was specifically expressed in spike. In contrast, *HvHPT1* was universally transcribed in root, leaf, stem, and spike. Further qRT-PCR analyses (Fig. 3B) showed that *HvHPT2* was specifically expressed in husk, whereas *HvHPT1* and *HvHGGT* were highly transcribed in embryo and endosperm, respectively. Moderate transcription of *HvHPT1* was also detected in husk. Transgenic overexpression in rice (Fig. 3C) showed that *HvHPT2* was indeed transcribed in husk. Protein subcellular location analyses showed that HvHPT2 was targeted to chlorophyll specifically, similar with that observed for HvHPT1 and HvHGGT (Fig. 3D).

Next, *HvHPT2* was overexpressed in barley “Golden Promise” which lacks *HvHPT2*. In the transgenic lines, qRT-PCR showed that the transcription of *HvHPT2* in leaf was about 20~35 times that in grain (Fig. 3E). In contrast, no expression of *HvHPT2* could be detected in the wild type (Fig. 3E). Vitamin E content measurement (Fig. 3F, G) showed that α-tocopherol was the major form of tocopherol in leaf tissue. Instead, comparable levels of α- and γ- tocopherol were accumulated in the grain. Compared to wild type, *HvHPT2* transgenic lines accumulated much higher amount of α-tocopherol in both leaf and grain, suggesting that *HvHPT2* is mainly functional in α-tocopherol production, similar as *HvHPT1* [43].

In accordance with HPLC profile, quantitative analysis confirmed that α-tocopherol was the predominant form of tocopherols in both wild-type and transgenic lines, accounting for 95–97% and 65–76% of total tocopherols in leaf (Fig. 3H) and grain (Fig. 3I), respectively. In contrast, γ-tocopherol only accounted for 2–4% in leaf and 20–30% in grain. In addition, β- and δ-tocopherol contents were low in all target samples. Compared to wild-type barley, α-tocopherol in *HvHPT2* transgenic lines increased significantly by 20–111% and 38–60% in leaf and grain, respectively. Furthermore, γ- and β-tocopherol also increased by about 2-fold in the leaf of transgenic lines, despite their low content. Noteworthy, no significant change for γ- and δ-tocopherol was observed in transgenic grains in comparison with wild type. In terms of total tocopherol content, the three *HvHPT2* transgenic lines displayed on average 36% and 55% increase in grain and leaf, respectively (Fig. 3J, Additional file 1: Table S4).

### Comparative gene retention analyses revealed a gene dosage constraint effect

**Fig. 4.**
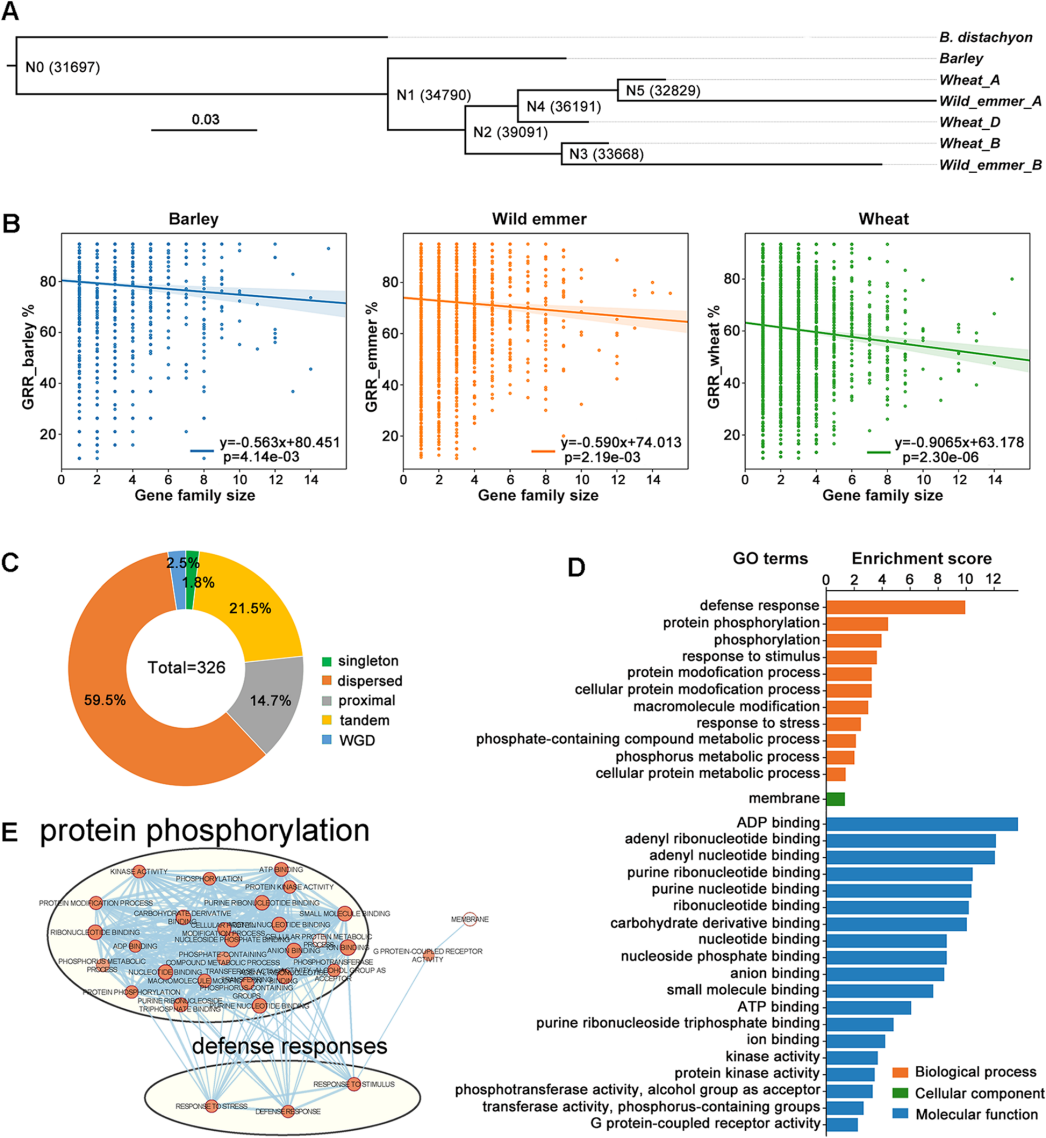
Phylogeny-based pangenome gene retention rates analyses and gene ontology enrichment analyses. **A** Reconstructed species phylogeny of barley, wild emmer, and bread wheat based on orthologous gene classification using OrthoFiner software. The numbers of hierarchical orthologous group (HOG) identified at each node were labelled. *B. distachyon* was included as outgroup. **B** Scatter plot displaying the correlation between mean GRR for each OG and gene family size (number of HOG in each OG) in barley, wild emmer, and bread wheat. **C** Pie chart displaying the duplication types of the identified 326 *HPT2-pattern-like* genes. **D** GO enrichment analyses of the identified *HPT2-pattern-like* genes displaying deceased GRR from barley to wild emmer to bread wheat. Enrichment score was calculated as −log_10_(*p-value*). **E** Gene network displaying the enriched biological pathways for the *HPT2-pattern-like* genes. Node size and color correspond to number of genes and significance level, respectively. Node edge (blue line) indicates number of overlapped gene

The potential correlation of the retention rates of *HPT2* with gene dosage constraint (functional redundancy) and environmental selection provided a valuable opportunity to investigate the gene retention process following gene duplication. To gain more insights into this important evolutionary process, we expanded gene retention analyses to whole genome scale and searched for genes with similar retention pattern with *HPT2* (defined as *HPT2-pattern-like* genes). Orthologous genes (Additional file 6) across barley, wild emmer, and bread wheat were inferred using an accurate phylogeny-based algorithm implemented by OrthoFinder program [51] using *B. distachyon* as outgroup species. As shown in Fig. 4A, the program reconstructed the species tree correctly. A total of 31,697, 34,790, and 39,091 hierarchical orthologous groups (HOG) at nodes N0, N1, and N2. The HOGs identified at node N1 covering the three target species (barley, wild emmer, and bread wheat) were selected for gene retention analysis.

Gene retention rate (GRR) of each HOG genes in 20 barley pangenome lines, 20 representative wild emmer lines, and 15 bread wheat pangenome lines (excluding the *Triticum spelta* line PI190962 in Fig. 2B) were calculated (Additional file 7). Linear correlation analyses showed that GRR is negatively associated with orthologous group numbers in the target lines of barley (−0.563), wild emmer (−0.590), and bread wheat (−0.907) (Fig. 4B), suggesting that genes from more expanded families are more likely to be lost. This finding supports the effect of gene dosage constraint on gene retention. Moreover, the negative correlation slopes seem to increase from barley (−0.563) to wild emmer (−0.590) to bread wheat (−0.907), suggesting that the gene dosage constraint effect tends to increase along with ploidy level.

### Genome-wide identification of *HPT2*-pattern-like genes and functional analyses

Based on GRR calculation, 315 orthologous HOGs containing 326 *HPT2-pattern-like* genes (barley as reference, Additional file 8) were identified to follow GRR_barley > GRR_emmer > GRR_bread wheat, representing a pool of candidate genes under ongoing gene fixation process and potentially affected by gene dosage constraints.

Depending on different genetic mechanisms, gene duplicates can be classified into WGD/segmental, tandem, proximal, and dispersed duplications. Gene duplication analyses (Fig. 4C, Additional file 9) showed that *HPT2-pattern-like* genes mainly result from small-scale duplication events, the highest being dispersed duplication (59.5%), followed by tandem duplication (21.5%) and proximal duplication (14.7%). In contrast, singleton genes and genes resulted from WGD event each account for 1.8% and 2.5%, respectively. These results suggest that small-scale duplications play a dominant role for genes under gene fixation.

To gain biological insights into the *HPT2-pattern-like* genes, GO enrichment analyses were performed (Fig. 4D, Additional file 10). At the molecular function (MF) level, the target genes were found significantly enriched with GO terms involved in various molecule binding functions (ADP-binding, nucleotide binding, ATP-binding, carbohydrate derivative binding, anion binding, small molecular binding), followed by kinase activity and receptor activity. Majority of these enriched molecular functions were related to molecular binding and interaction. At the biological pathway (BP) level, these enriched GO terms were found to be significantly related to defense response, protein phosphorylation, phosphorylation, response to stimulus, and response to stress suggesting that *HPT2-pattern-like* genes may be closely associated with plants’ environmental adaption. At the cellular component (CC) level, *HPT2-pattern-like* genes were found significantly enriched with GO terms related to membrane component, consistent with an active role for membrane protein in plants’ defense responses. Further gene network (Fig. 4E) analyses showed that *HPT2-pattern-like* genes are significantly enriched with two major biological processes: protein phosphorylation and defense responses, which are closely linked with each other.

### *HPT2*-pattern-like genes displays relaxed selection and are under varying degrees of positive selection

**Fig. 5.**
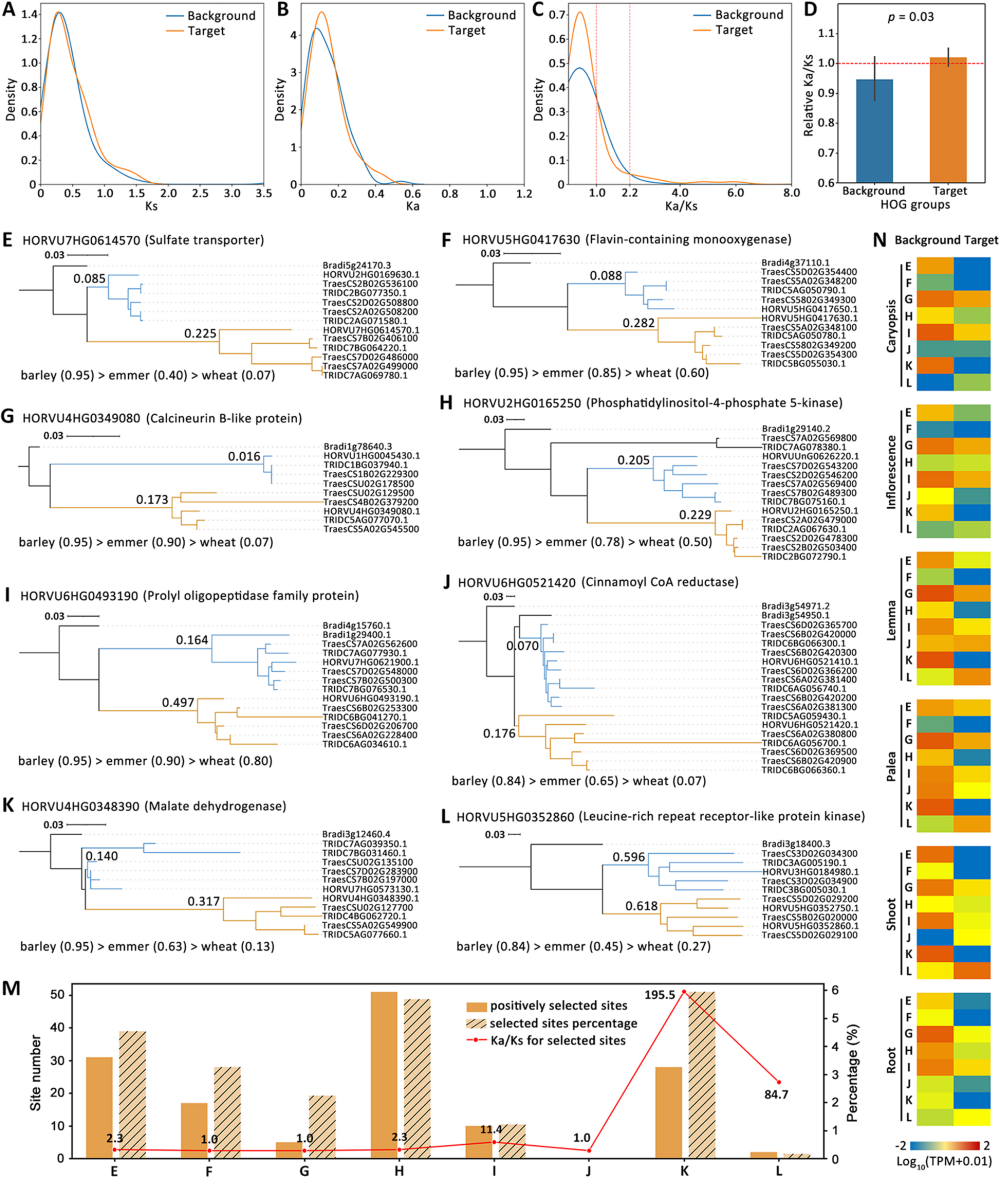
Natural selection, evolutionary pattern, and transcriptional divergence of *HPT2*-pattern-like genes. Comparing the distribution patterns of *Ks* (**A**), *Ka* (**B**), and *Ka/Ks* (**C**) for the target 326 *HPT2*-pattern-like genes (brown color) of decreasing retention rates in barley, wild emmer, and bread wheat pangenome lines to their homologous background genes (blue color). **D** Comparing the relative *Ka/Ks* value of target genes (brown) and background genes (blue) to the mean *Ka/Ks* for each orthologous group. A relative Ka/Ks value higher than 1 indicates elevated *Ka/Ks*. **E–L** Display 8 example gene trees from the 326 target genes exhibiting a gene dosage effect. Corresponding barley gene ID, annotated gene function, *Ka/Ks* values (labelled above branch line) for target (brown) and background (blue) HOG groups, and the calculated gene retention rates (below gene tree) for target genes were presented. Barley gene ID were truncated by removing “MOREX.r2”. **M** Bar chart displaying the identified amino acid sites under positive selection for genes in **E–L**. The number, percentage, and Ka/Ks value of the positively selected sites for each gene were displayed. **N** Displays the transcriptional divergence of background (left) and target (right) barley genes based on transcriptome data in six barley tissues in Morex cultivar. Caryopsis: developing grain 5DPA; inflorescence: 1–1.5 cm; lemma: 42 DPA; palea: 42 DPA; shoot: 10 cm stage; root: 28 DPA

To characterize the natural selection pressure of *HPT2-pattern-like* genes, *Ka*, *Ks*, and *Ka*/*Ks* for the *HPT2-pattern-like* genes (target) and their closest homologous genes (putative parental genes, defined as background) were calculated (Additional file 11). Results (Fig. 5A) showed that the target and background genes have similar *Ks* distribution profiles with overlapping peak values, indicating that they have experienced similar evolution time. In contrast, the *Ka* for the target genes peaked at a higher value than that for the background genes (Fig. 5B), implying an elevated selection pressure. Indeed, when *Ka*/*Ks* was calculated and compared, more target genes was found in the *Ka*/*Ks* > 2.2 range, indicating a higher level of positive natural selection (Fig. 5C). In addition, a higher percentage of target genes were also observed for the *Ka*/*Ks* < 1.0 range, which needs to be further examined on the basis of individual gene. Compared to the mean *Ka*/*Ks* of each orthologous group, the target genes (Fig. 5D) displayed relatively higher *Ka*/*Ks* values, supporting a relaxed selection pressure. In contrast, the background genes exhibited significantly (*p* = 0.03) lower *Ka*/*Ks* values, indicating a stronger purifying selection.

To detect potential positive selection, we randomly selected 8 *HPT2-pattern-like* genes (Fig. 5E–L) and drew the phylogenetic trees using *B. distachyon* gene as outgroup (Additional file 12). In terms of gene functions, most of these 8 genes can be related to environmental adaptation: transporter (Fig. 5E), disease resistance kinase (Fig. 5H, L), antioxidant biosynthesis (Fig. 5F, J), calcium sensor (Fig. 5G), regulating protein kinase, oxidoreductase (Fig. 5K), and oligopeptidase (Fig. 5I). *Ka*/*Ks* calculation for the target (brown) and background (blue) lineages (Fig. 5E–L) showed all target lineages display higher *Ka*/*Ks* than their parental genes, suggesting generally relaxed selection pressure. All 8 target genes have *Ka*/*Ks* value below 1.0, indicating a functional constraint. When branch-site selection model was applied, results (Fig. 5M) showed that positive selection was detected in 7 out of the 8 target genes, with the exception of gene J. The percentage of positively selected sites ranges from 0.16% in gene L to 5.94% in gene K, implying varied levels of positive selection.

For transcriptional divergence analysis, transcriptional data for target and background genes in six barley tissues (Caryopsis: 5DPA; Inflorescence: 1–1.5 cm; Lemma: 42 DPA; Palea: 42 DPA; Shoot: 10 cm stage; Root: 28 DPA) were retrieved and compared. The transcription of the 8 target genes clearly varied from that for the background genes (Fig. 5N). Overall, the background genes tend to be actively transcribed in multiple tissues, while the target genes generally have decreased expression in the six tissues and more likely to be transcribed specifically in some tissues.

### Comparative gene retention analyses in barley and bread wheat reveals a species-specific selection pattern

**Fig. 6.**
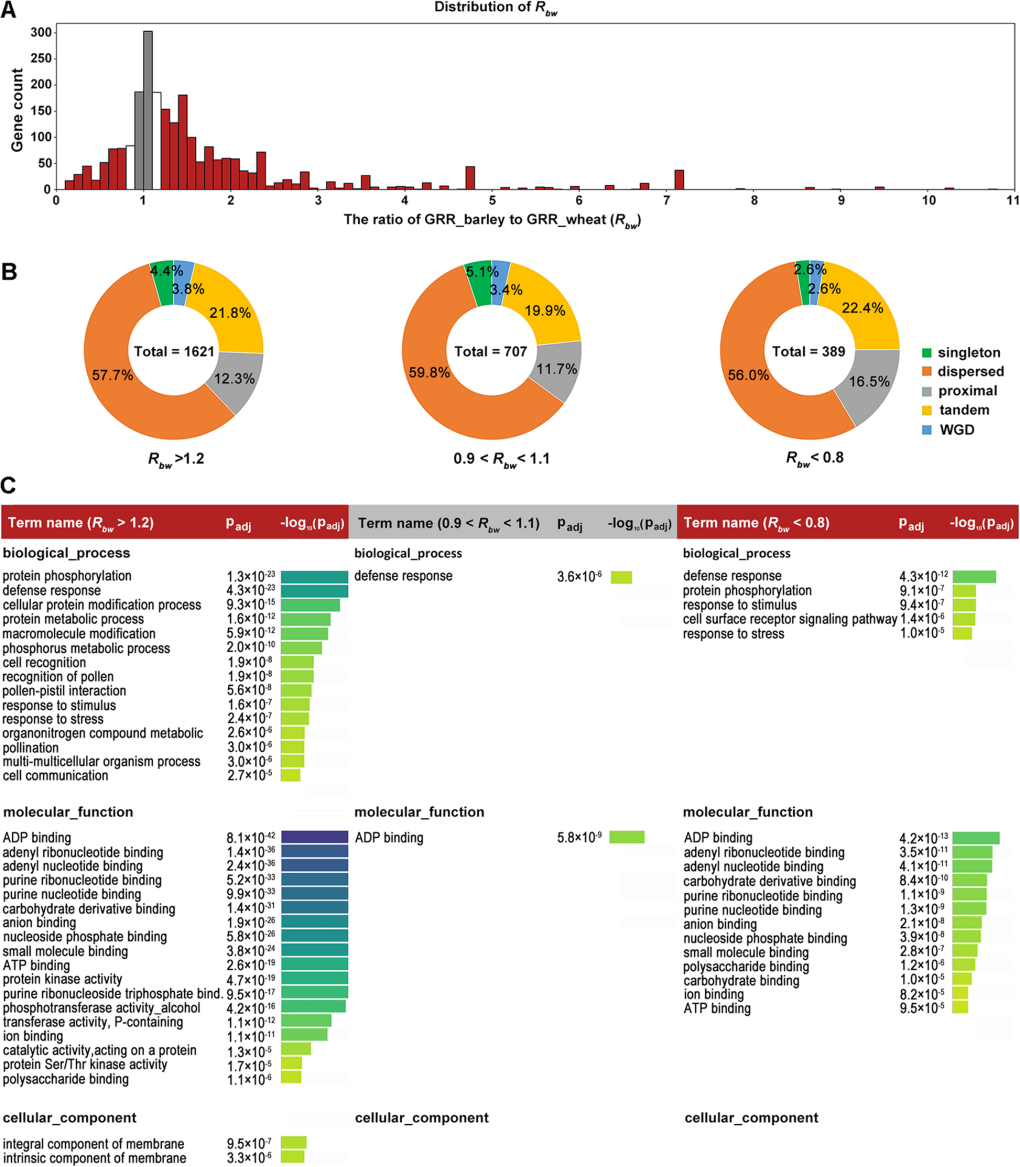
The species-specific selection pattern of gene retention in barley and bread wheat pangenome lines. **A** Distribution of the ratio (*R*_*bw*_) of *GRR_barley* to *GRR_wheat*. The target genes were divided into three categories: *R*_*bw*_ ≥ 1.2 (red color), 1.1 > *R*_*bw*_ > 0.9 (gray color), and *R*_*bw*_ ≤ 0.8 (red color) for enrichment analyses. **B** Donut chart displaying the distribution of gene duplication types for each gene category. The total gene number of each category was labelled at the center. **C** Comparative GO enrichment analyses for the three gene retention categories in barley and bread wheat pangenome lines. Each category implies different selection pressure acting on gene retention. A threshold of 1e−5 was used to filter the enriched GO terms. No significantly enriched cellular component item was found for the neutral and wheat preferential gene categories

Species-specific gene family evolution is a common observation in plants. To investigate if gene retention may be affected by species-specific selection pressure, we selected barley and bread wheat as example species and divided the partially retained genes into 3 categories: GRR_barley > GRR_wheat (barley preferential), GRR_barley = GRR_wheat (neutral), and GRR_barley < GRR_wheat (bread wheat preferential). To reduce potential confounding effect during GRR calculation, we applied a more stringent criteria: by defining *R*_*bw*_ = GRR_barley / GRR_wheat, the three gene categories were selected as *R*_*bw*_ ≥ 1.2 (barley preferential), 1.1 > *R*_*bw*_ > 0.9 (neutral), and *R*_*bw*_ ≤ 0.8 (bread wheat preferential) (Fig. 6A). Due to a higher ploidy of bread wheat (hexaploid) than that of barley (diploid), we expect a relaxed gene dosage constraint in bread wheat, thus a relatively lower GRR in bread wheat. Therefore, a lower number of gene should be identified for *R*_*bw*_ ≤ 0.8 than that for *R*_*bw*_ ≥ 1.2. Indeed, a total of 1621, 707, and 389 genes were obtained for the above 3 categories, respectively (Fig. 6A,B). The number of genes preferentially retained in barley is over 4 times of that preferentially retained in bread wheat. The higher number of barley preferential genes than wheat is consistent with a gene dosage constraint hypothesis: Since the bread wheat genome is hexaploid while the barley genome is diploid, for each orthologous gene group, wheat generally has a higher gene dosage than barley. The higher gene dosage/redundancy in wheat would result in a lower gene constraint on individual gene duplicate. Therefore, newly duplicated genes are more likely to be lost than that in barley due to a varied selection constraint. Moreover, the gene dosage effect on gene retention rates is also consistent with our results in Fig. 4B, which suggests that genes from a large gene family are more likely to be lost. Genomic data from additional species in *Triticeae* can be used to further verify our hypothesis. In addition to gene dosage effect, other unknown factors that may also contribute to biased gene retention rates between barley and bread wheat, which requires further investigation.

To explore if the gene duplication type may vary for the 3 gene categories, their duplication type profiles were determined. Results (Fig. 6B) showed that the 3 gene sets displayed a similar gene duplication type composition, which is dominated by dispersed duplicates (56.0~59.8%), followed by tandem duplicates (19.9~22.4%) and proximal duplicates (11.7~16.5%), sequentially. WGD and singleton duplicates account for 2.6~3.8% and 2.6~5.1%, respectively. These observations suggest that gene duplication type is independent from species. The partially retained genes in barley and bread wheat genomes have resulted from similar gene duplication mechanisms.

Next, functional enrichment analyses was performed for each gene category and compared. For the *R*_*bw*_ ≥ 1.2 (barley preferential) group, the highest enrichment significance (Fig. 6C, left) was observed. Overall, the enriched GO terms for *R*_*bw*_ ≥ 1.2 resemble that identified for the 326 *HPT2-pattern-like* genes at the MF, BP, and CC levels. Genes with various molecular binding and protein kinase functions, and related to protein phosphorylation and defense response were among the highest enriched GO terms. In contrast to the *HPT2-pattern-like* genes, a significant number of GO terms related to pollen recognition, cell recognition, pollen-pistill interaction, and poolination were identified. For the *R*_*bw*_ ≤ 0.8 (bread wheat preferential) group (Fig. 6B, right), a significant number of enriched GO terms were also identified. Compared to *R*_*bw*_ ≥ 1.2, *R*_*bw*_ ≤ 0.8 displayed some overlapped GO terms such as protein phosphorylation, plant defenses, and responses to stimulus. These results indicated that the bread wheat preferential genes (*R*_*bw*_ ≤ 0.8) were also under significant selection pressure, albeit at a relatively lower significance level. In contrast to *R*_*bw*_ ≥ 1.2 and *R*_*bw*_ ≤ 0.8, barely no GO term was found to be enriched for the 1.1 > *R*_*bw*_ > 0.9 (neutral) group at the significant level, except that ADP-binding and defense response were weakly enriched (Fig. 6B, middle), indicating that the 1.1 > *R*_*bw*_ > 0.9 group were more likely under neutral selection and may play a less significant role in plant-specific evolution. In summary, gene enrichment analyses revealed a stronger selection signal in the barley preferential group, followed by the wheat preferential group, and the least in the neutral group. This observation is consistent with a putative species-specific selection and gene dosage effect theory, whereby the wheat preferential gene group may represent the species-specific selection effect only, while the barley preferential gene group results from the combined species-specific selection and gene dosage effect.

## Discussion

The process leading to gene fixation following duplication is critical to understand how genomes evolve and thus has attracted great interest from evolutionary biologists. Various models and hypotheses [5, 33] have been proposed to explain how gene duplicate is retained following duplication. One notable limitation for earlier studies [23, 25, 27, 31, 52] is that they were all based on gene duplicate analyses in single reference genome. Gene duplicates present in individual reference genome are mainly caused by ancient ploidy or other long-term duplication events [23]. Therefore, the observed gene retention pattern may not reflect directly how gene duplicates can be retained. Since the gene fixation process of gene duplicates occurs in the context of a population, we reason that analyzing the gene retention pattern at the population level would enable us to identify gene duplicates under gene fixation selection.

### Population-based gene retention analysis captures gene evolution process via duplication and reveals the effect of gene dosage constraint on gene retention

Based on comparative gene retention analyses in barley, wild emmer, and wheat pangenome lines, we dissected a convincing example of an evolutionary lineage *HPT2* caught in the middle stage of gene fixation. Clear evidences of gene duplication, partial gene retention, and functional constraints support *HPT2* being in the middle stage of gene fixation can be drawn from. The loss of *HPT2* in some lines of the pangenome lines is in accordance with classical population theory that the most likely fate of gene duplicate is gene loss [16, 17]. Our observation of *HPT2* being duplicated in the common ancestor of *Triticeae* and *Avenidae* suggests that gene loss can persist over a long timescale, but is not restricted to the early stage following duplication [52, 53]. Although presence/absence variation (PAV) mutation is commonly observed in previous studies [39, 42, 54], what distinguishes *HPT2* from a common PAV is that its retention is associated with *HPT1* gene dosage, potentially due to their functional redundancy. Our assumption of functional redundancy between *HPT1* and *HPT2* is verified at both gene transcriptional and functional levels. A higher copy number of homologous genes in wild emmer and bread wheat would make *HPT2-pattern-like* genes functionally more redundant, thereby leading to a higher chance of gene loss. The gene dosage hypothesis agrees with previous studies [52, 53, 55, 56] based on single reference genome, which suggest gene dosage constraints as one potential factor affecting gene retention following gene duplication. In this study, the effect of gene dosage constraint on gene retention is further corroborated by the correlation analyses between GRR and gene family size, independently in barley, wild emmer, and bread wheat pangenome lines. The negative correlation between GRR and gene family size support that gene duplicates from more expanded gene family are more likely to be lost. Interestingly, we found that this gene dosage constraint effect on gene retention tends to increase with genome ploidy level, which also makes sense considering the potentially higher genetic redundancy in higher ploidy genomes.

Accurate detection of genuine orthologous gene groups across multiple species is critical for the comparative gene retention analyses in this study. Compared to traditional sequence similarity-based approaches, the phylogeny-based orthology inference by OrthoFinder has the advantages of higher accuracy and high throughput at the whole genome scale [51]. A recent study [57] reported a novel collinearity-incorporating method for homology inference in *Triticeae*. However, considering the large amount of transposable element-mediated gene duplication events in *Triticeae* [58], the collinearity-incorporating method is less preferred in this study where most of the target *HPT2-pattern-like* genes were found to be SSDs. The innovative combination of phylogeny-based orthologous gene inference and population-based gene retention analysis enables us to identify 326 *HPT2-pattern-like* genes at whole genome scale. We argue that these *HPT2-pattern-like* genes represent a pool of candidate genes under ongoing gene fixation and share two common features: firstly, duplication occurred in the common ancestor of barley, wild emmer, and bread wheat; secondly, biased retention across species. This gene pool provides a valuable opportunity to characterize the evolution profile of the gene retention process following duplication. For example, we observed apparently relaxed selection pressure among *HPT2-pattern-like* genes. This finding is consistent with previous theoretical prediction that duplicated genes will undergo immediate relaxed selection due to reduced functional constraints [17, 59, 60]. In particular, SSDs seem to exhibit a higher level of relaxed selection than WGDs [60]. Indeed, most of the *HPT2-pattern-like* genes were determined as SSDs in this study. There are increasing evidence showing that relaxed selection, coupled with positive selection, plays a critical role for the rapid evolution of phenotypic plasticity [61–63]. Our study is also corroborated by a recent study which reports that enhanced mutation rate and relaxed selection are associated with gene loss in microorganisms [64]. In addition to relaxed selection, we also found positive selection and transcriptional divergence are prevalent among *HPT2-pattern-like* genes, which are consistent with previous observations and predictions [4, 65–69]. The highly similar evolution profile for *HPT2* and *HPT2-pattern-like* genes support *HPT2-pattern-like* genes being in the middle stage of gene fixation. Our analyses prove that population-based gene retention analyses may serve as a novel and effective approach to study gene evolution via duplication in a “real-time” manner.

### Environmental selection plays a clear role on gene retention following duplication

In addition to gene dosage constraint, we found that environmental selection is another impacting factor affecting the retention of *HPT2*. We found additional cis-regulatory elements related to low-temperature responses in the putative promoter region of *HvHPT2* (Additional file 1: Table S5). The elevated retention rate of *HPT2* in Tibetan barley may be attributed to its association with low-temperature adaption. This type of allele frequency bias within a subpopulation has been widely identified as a signature of natural selection [70–72]. In addition, the presence and absence of *HPT2* in wild barley lines collected from the two abutting slopes of EC [46], respectively, provides direct evidence that *HPT2* may be involved in environmental adaption to microclimates. The retention of *HPT2* in wild barley in the cool and humid NFS slope is consistent with the proven function of tocopherols in low-temperature responses [45, 50]. In addition to barley, the retention of *HPT2* in bread wheat should also be subjected to environmental selection, which can be verified by comparing different wheat populations under different environmental conditions. However, the presence of 3 copies of *HPT1* in bread wheat genome would make *HPT2* less important in bread wheat than that in barley, resulting in lower selection pressure on bread wheat *HPT2*. Therefore, we predict that the variation of *HPT2* retention rates in different wheat populations would be less prominent than that in barley. This prediction would also apply to *HPT2* in wild emmer. Gene retention analyses in wild emmer and bread wheat population are hindered by the lack of proper wild emmer WGS data and by the lack of *HPT2* in bread wheat reference genome Chinese Spring. It would be interesting for future studies to verify our prediction.

Environmental selection on gene retention is also supported by the detection of positive selection, which has been commonly related to plant-environmental interaction and stress responses [68, 69]. Positive selection was detected for *HPT2* and 7 out of the 8 randomly selected *HPT2-pattern-like* genes, suggesting that environmental selection should be prevalent among genes under gene fixation. Consistently, gene functional enrichment analyses suggest that the *HPT2-pattern-like* genes are over-represented with plant defense responsive function. A recent study found that a cluster of SSDs genes shared by core *Pooideae* species coincided with their adaption to cooling conditions [73]. This is consistent with our findings with *HPT2-pattern-like* genes, which are dominated by SSDs and are shared by barley, wild emmer, and bread wheat. In this study, *HPT2-pattern-like* genes were identified to be in the middle stage of gene fixation, which are partially retained in the populations. Gene PAV mutations have also been shown to have important impact on phenotypic traits and stress tolerance [54, 74, 75]. Many of the *HPT2-pattern-like* genes have annotated gene functions that are closely related to plants’ stress resistance, such as those exemplified in Fig. 5: transporter proteins, disease resistance kinase, antioxidant biosynthesis, calcium sensor regulating protein kinase, and oxidoreductase. Given that the *HPT2-pattern-like* genes were selected based on their gene dosage effect, the enrichment of stress and defense-related genes in *HPT2-pattern-like* genes also suggests that these genes under stronger environmental selection may more likely display a gene dosage effect. Take *HPT2* for example, firstly, the gene dosage (functional redundancy) effect results in the decreasing retention rates of *HPT2* in barley (*HPT1*+*HPT2*), wild emmer (2*HPT1*+*HPT2*), and wheat (3*HPT1*+*HPT2*), caused by the relaxed selection constraint as gene dosage increases. Secondly, the association of *HPT*’s (vitamin E) with environmental adaption adds an additional selection pressure, which would make *HPT2* in barley more important, while *HPT2* in wheat is less affected due to the presence of 3 *HPT1*s. Thus, the gene dosage effect on *HPT2* retention tends to be amplified. This theory can partly explain the enrichment of plant defense function in the 326 *HPT2-pattern-like* genes.

### Biased gene retention pattern in terms of gene function

We found that *HPT2-pattern-like* genes are significantly over-represented with molecular-binding and interaction functions. Despite of using different data and approach, our findings are in striking agreement with previous reports that gene duplicates encoding transcription factors and protein kinase are preferentially preserved after WGD in *Arabidopsis*, rice, and yeast [23, 25, 31]. The biased retention of gene duplicates involved in molecular interaction (various molecular binding such as transcriptional factors) is consistent with previous gene dosage balance hypothesis [28, 31], which states that gene function in a molecular complex displays higher gene dosage effects. Indeed, the *HPT2-pattern-like* genes were selected being potentially affected by gene dosage constraint. In addition, it has been reported that genes resulted from SSDs were enriched for membrane proteins and functions in stress tolerance (and under-presented for transcription factors and DNA/RNA binding functions), displaying a reciprocal relationship with that observed for WGDs [27, 28]. We found that *HPT2-pattern-like* genes (mainly SSDs) were enriched for membrane proteins and defense responses functions, similar with the observation for tandem duplications in a previous study [27]. Our findings are corroborated by a recent comparative study in 141 plant genomes, which showed that SSDs such as tandem and proximal duplications have evolved toward biased functional roles in plant self-defense [37]. In this study, we found that *HPT2-pattern-like* genes are mainly resulted from SSDs, instead of WGDs. Our findings tend to support an inter-twined scenario of previous studies: on the one hand, *HPT2-pattern-like* genes (mainly SSDs) resemble previous WGDs in enrichment for molecular binding, transcription factors, and protein kinase [23, 25, 31]; on the other hand, *HPT2-pattern-like* genes are also enriched in membrane and defense responsive proteins, similar with the tandem duplications in previous studies in *Arabidopsis* and rice [27, 28]. The enrichment of transcription factor and molecular binding proteins in these SSDs may be attributed to *HPT2-pattern-like* genes being filtered for genes under potential gene dosage effect, which agrees with previous suggestion that genes involved in subunit-subunit interactions tend to be under higher gene dosage constraints [31]. Noteworthy, a recent study [58] supported a recent burst of gene duplications (RBGD) in *Triticeae*, mainly by transposable element-mediated SSDs. Functional enrichment analyses found that protein dimerization activity, xylan metabolic process, catalytic activity, and nucleobase-containing compound metabolic process were enriched among RBGD genes, which seem to vary from the functional profile for our identified *HPT2-pattern-like* genes [58]. The enrichment of plant defense responses and molecular binding functions in *HPT2-pattern-like* may reflect the gene dosage selection effect, which is likely to enhance among partially retained genes related to environmental adaption.

### Species-specific selection affecting gene retention

In this study, we found that selection on gene retention follows a species-specific pattern. Although with some variations, both barley and bread wheat preferential genes overlap on protein phosphorylation, defense, and stress responsive functions, whereas the neutrally selected genes displayed no clear enrichment. The detection of overlapped functions in bread wheat preferential genes was not expected, which reflect a different type of selection pressure from gene dosage constraints. We define this unknown selection pressure as species-specific selection. The species-specific selection hypothesis is in agreement with an earlier study, which states that the evolution of gene duplicate depends on its duplication mechanisms (WGD and SSD) but, most importantly, on species [60]. Based on evolutionary dynamics analyses on gene paralogs in 8 plant species, the authors concluded that species-specific selection plays dominant role in determining gene retention [60]. We speculate that the preferentially retained genes in barley may be influenced by combined effects of gene dosage constraint and species-specific selection, which well-explains more genes being preferentially retained in barley. Instead, bread wheat preferential genes may be related to species-specific selection only. The species-specific selection hypothesis may contribute to species-specific gene duplication events in various plant genomes [7, 14, 37]. Indeed, species-specific gene family expansion via duplication, particularly SSDs have been shown to play an important role in plants’ stress responses and disease resistance [76–78].

### *HPT2*-pattern-like genes provide new opportunity for crop improvement

Among monocot plants, the *Poaceae* family contains some of the most important crops such as bread wheat, rice, maize, barley, and oat, which are the major food sources for human being [79]. In these cereal species, genetic engineering and crossing have been used as an important breeding approach to improve grain yield and nutrient content under various stress and changing climate conditions [80, 81]. Here, we propose that the *HPT2-pattern-like* genes identified in this study represent an interesting list of candidate genes for future crop improvement in cereal plants. Compared to a common list of PAV or copy number variation genes as reported in previous studies [39, 54], the *HPT2-pattern-like* genes identified in this study were specifically selected for their biased retention in barley, wild emmer, and bread wheat, which reflects a long-term environmental selection effect. Thus, the introduction of *HPT2-pattern-like* genes via either genetic engineering or crossing may have great potential to improve crop performance under various environmental challenges.

The potential application of *HPT2-pattern-like* genes for crop improvement can be exemplified by the transgenic overexpression of *HvHPT2* in barley. As the major lipid soluble antioxidant synthesized in green plants [82], vitamin E not only plays a crucial role in plant growth [83] and various stress tolerance [84], but also forms an essential component in human diet with numerous nutritional and medical values [85, 86]. A sufficient intake of vitamin E can help to prevent neurological disorders and chronic diseases, such as atherosclerosis, cataracts, and cancers, and maintain the healthy development of various tissues and organs, including brain, nerves, muscle and bone, skin, bone marrow, and blood [85, 86]. Due to the importance of vitamin E for both plants and humans, genetic engineering to improve vitamin E content has become an important target in crop breeding [87]. Flagship crop species like bread wheat [88], barley [43, 89], maize [44], sorghum [90], rice [91–94], and soybean [95–100] have been the focus of vitamin E genetic engineering. In this study, we showed that the overexpression of *HvHPT2* can significantly increase α-tocopherol content in leaf and grain, proving *HvHPT2* as an effective candidate gene for future genetic engineering and breeding to improve vitamin E content in barley. Future study is necessary to identify superior *HvHPT2* allele with higher vitamin E production and cold-responsive transcription, particularly those conserved in Tibetan barley. In addition to barley, *HPT2s* from *T. intermedium*, wild emmer, bread wheat, and *A. eriantha* also deserve attention to explore their potential application for improved vitamin E content and stress tolerance in crop breeding. We speculate that *HPT2* should also be present in other *Triticeae* and *Avenideae* plants such as *S. cereale*, *T. urartu*, *T. tauschii*, and *A. atlantica* despite its absence in their reference genomes. Genome sequencing in these species is needed to verify our hypothesis.

As exemplified by *HPT2*, the potential use of the identified 326 *HPT2-pattern-like* genes in crop improvement can also be investigated. Special attention should be given to those genes encoding transcription factors and related to plant defense responses. In addition, genes encoding membrane proteins, most of which involve molecular interactions and display higher gene dosage balance effect, also deserves special attention. Indeed, *HPT2* itself also functions as a membrane-binding protein, critical for maintaining the integrity of cell membrane under various stresses [82]. In addition, the importance of membrane proteins such as leucine-rich repeat receptor kinase plant defense has been highlighted in many previous studies [101–103]. It should be pointed out that the biased gene retention rates for the 326 genes may be attributed to the gene dosage effect but may also result from other factors such as random occurrence. The general gene functional and selection signals were detected by the overall gene enrichment and natural selection analyses, but did not verify that all of these 326 genes resemble the case of *HPT2*. The value of the identified gene pool is that it provides an enriched pool to identify *HPT2-pattern-like* genes. The factors affecting the retention rate, function, and evolution pattern of each gene still needs to be examined individually.

## Conclusions

Based on comparative gene retention analysis across barley, wild emmer, and bread wheat pangenome lines, we dissected a convincing example of *HPT2* caught in the middle stage of gene fixation. We innovatively combined phylogeny-based orthology inference with population-based gene retention analysis, and identified 326 *HPT2-pattern-like* gene duplicates under gene fixation selection potentially affected by a gene dosage effect. Gene retention, natural selection, and functional enrichment analyses support gene dosage constraint, environmental adaption, and species-specific selection as three potential factors that determine gene retention process following duplication. Genes under stronger environmental selection more likely display a gene dosage effect in term of gene retention. Our analyses prove that population-based gene retention analyses may serve as a novel and effective approach to study gene evolution via duplication. Our findings will greatly improve our understanding of genome evolution in plants and other living organisms.

## Methods

### Identification of homologous *HPT* genes in Poales genome

UbiA domain profile (PF01040) was downloaded from Pfam database (http://pfam.xfam.org/). Putative HPT genes were identified using hmmscan in HMMER package (http://hmmer.org/). Annotated protein sequences for barley reference genome Morex (V2) and wild emmer (V2) were downloaded from IPK (10.5447/IPK/2019/8) and GrainGenes (https://bread wheat.pw.usda.gov/GG3/), respectively. Genomic datasets for other species were retrieved from either Phytozome V12 (https://phytozome-next.jgi.doe.gov/) or EnsemblPlant release-54 (https://plants.ensembl.org/index.html) databases. The presence/absence of *HPT2* in wheat pangenome assemblies were validated by tblastn using the amino acid sequence of TraesLAC2B03G00984720.1 from wheat cultivar Lancer. The tblastn results against 15 wheat genomes were accessible at https://plants.ensembl.org/Triticum_aestivum_lancer/Tools/Blast/Ticket?tl=d6dR15Gh5DUEuDRG.

### Phylogenetic tree construction

Sequence alignment was performed using Muscle (8 iterations) [104]. Amino acid sequences were used for NJ phylogeny reconstruction in MEGA7.0 [105] using p-distance substitution model. CDS sequences were used for ML tree construction using IQ-TREE (V1.6.12) [106] with MG+F1X4+G4 substitution model (lowest BIC score). Branch support was calculated by 1000 times bootstrapping and SH-like approximate likelihood ratio test. Tree annotation was performed using Figtree software (v1.4.3, http://tree.bio.ed.ac.uk/software/figtree). Calibrated species phylogeny was obtained at http://www.timetree.org/ website. *HvHPT2* SNP data for barley pangenome lines (10.5447/ipk/2020/24) and Tibetan barley lines (https://edal.ipk-gatersleben.de/) were downloaded and used for ML tree construction using IQ-TREE with MGK+F1X4+G4 substitution model.

### Gene presence and absence analyses

SNP data for barley pangenome lines (177 SNPs) [39] and Tibetan barley (136 SNPs) [107] was used to screen *HPT2* presence/absence based on the missing genotype calls. For wild barley lines at EC in Israel, data at http://146.118.64.11/BarleyVar was searched. For orthology inference in barley and bread wheat pangenomes, Get_Homologues-EST was used following closely to the online manual (http://eead-csic-compbio.github.io/get_homologues/manual-est/). OrthoMCL (OMCL) algorithm was used for clustering with an inflation index of 1.5 as the cuttoff. Morex and Chinese Spring were used as the control for barley and bread wheat, respectively. For wild emmer, pangenome data were downloaded in the NCBI Sequence Read Archive database (accession IDs listed in Fig. 2B). Read adapters were removed using Trimmomatic v.0.36 [108]. Alignment was performed using bwa-mem [109] with default settings and the results were used to call gene deletions using CNVnator v.0.33 [110] based on read mapping depth with non-overlapping windows.

To validate the wheat genome annotation quality, BITACORA genome scan tool [111] was implemented on the OGs containing the selected HPT2-pattern-like genes. The hmmscan tool from the HMMER package (http://hmmer.org/) was used to identify conserved protein domain with E-value threshold of 1e−30. The corresponding hmm protein domain profile for each OGs and their protein sequences were used as input for BITACORA scanning using the same threshold of 1e−30. The results (Additional file 13) showed that no newly predicted gene for the target OGs could be identified, thereby confirming the reliability of the wheat genome annotation data.

### Natural selection tests

Natural selection pressure was assessed using codeml in PAML4.7 package [112]. CDS sequence alignment of *HPTs* was performed using Muscle [104]. Branch pattern specification was implemented using Treeview1.6.6 (http://taxonomy.zoology.gla.ac.uk/rod/treeview.html). *P*-value for likelihood ratio tests was calculated using Graphpad software (https://www.graphpad.com/quickcalcs/PValue1.cfm). For pair-wise *Ka*, *Ks*, and *Ka*/*Ks* calculation, the ParaAT tool [113] was used.

### RT-PCR analyses

RNA was extracted using EZNA Plant RNA following the manufacturer’s instructions (Omega, Norcross, USA). One microgram RNA was digested with a gDNA Remover kit and reversely transcribed into cDNA in ReverTra Ace qPCR RT Master Mix (Toyobo, Kyoto, Japan) in a 20-μL reaction volume. *HvACTIN* was used as reference gene. Three biological replicates and three technical replicates were included. All primers involved in RT-PCR analyses are listed in Additional file 1: Table S6.

### Vector construction, transformation, GUS staining, and subcellular localization

Morex cDNA of *HvHPT2* was subcloned into pH7FWG2.0 vector with *35S* promoter. Immature scutella (1.5–2 mm) of barley cultivar “Golden Promise” embryo were used for *Agrobacterium*-mediated transformation following previously described procedure [114].

*ProHvHPT2:GUS* containing 2.3-kb *HvHPT2* promoter was subcloned into destination vector pHGWFS7 containing *Egfp*:*GUS* by LR clonase reaction (Invitrogen, USA). Rice transformation for *ProHvHPT2:GUS* was performed by *Agrobacterium*-mediated co-cultivation approach. Regenerated plants were grown in a growth chamber. All primers involved in gene constructs are listed in Additional file 1: Table S6.

*35S:HvHGGT:GFP*, *35S:HvHPT1:GFP*, and *35S:HvHPT2:GFP* constructs were generated using Gateway cloning (Invitrogen, USA) and transformed into barley mesophyll protoplasts via PEG-mediated transfection, followed by incubation at 25°C in dark for 14 h before observation under a LSM 710 NLO confocal microscope (Zeiss, Jane, Germany).

T_1_ generation of *HvHPT2*-GUS transgenic lines were incubated in GUS staining solution and vacuum infiltrated for 10min, followed by incubation at 37°C for 2h. After staining, the samples were washed with 75% ethanol to remove chlorophyll and observed under a stereoscope. Original protoplast imaging files were deposited in Additional file 14.

### Vitamin E measurement

Vitamin E was extracted according to previously described method [43]. Agilent 1200 HPLC (Agilent Technologies) equipped with Phenomenex Kinetex F5 100A column (2.6 μm, 150 × 4.6 mm; Phenomenex) and G1321A detector was used for tocopherols and tocotrienols quantification, based on external standard curves using authentic compounds (ChromaDex).

### Phylogeny-based orthologous group inference

Protein sequences for barley (Morex V2), wild emmer (Zavitan WEWSeq v2.0), and bread wheat (Chinese Spring IWGSC refseqv2.1) were downloaded from 10.5447/IPK/2019/8 and https://bread wheat.pw.usda.gov/GG3/. Genomic data for *B. distachyon* (V2.1, https://phytozome-next.jgi.doe.gov/) was included as phylogenetic outgroup. Orthologous gene inference was performed using OrthoFinder [51] following the manual at https://github.com/davidemms/OrthoFinder.

### Transcriptome analyses

Transcriptome data of six barley tissues: Caryopsis: developing grain 5DPA; inflorescence: 1–1.5 cm; lemma: 42 DPA; palea: 42 DPA; shoot: 10 cm stage; root: 28 DPA, was retrieved from EORNA transcriptome database [115] (https://ics.hutton.ac.uk/eorna/index.html). For each sample, three biological replicates were included. The mean transcripts per million reads (TPM) value for each gene was used for heatmap generation based on log (TPM+0.01), 10).

### Gene duplication pattern and synteny analyses

MCScanX [116] was used for gene duplication and synteny analyses. Genome comparison was performed using the standalone NCBI-BLAST-2.2.29 (ftp://ftp.ncbi.nlm.nih.gov/blast/executables/blast+/LATEST/) with *E*-value threshold of 1*e*−5. The top 5 blastp hits were retained. Gene duplication type was determined using *duplicate_gene_classifier* program in MCScanX.

### Gene functional enrichment and network analyses

Pathway analyses were performed using g:Profiler (https://biit.cs.ut.ee/gprofiler) with barley as reference. To facilitate input for g:GO St, candidate barley genes (Morex, annotation version V2) were mapped to version V1 gene ID using blastp program (*E-value* 1*e*−10). Enrichment *P* values were estimated by hypergeometric distribution tests and adjusted by multiple testing correction using the g:SCS algorithm, with a threshold at 0.05. For gene network analysis, g:Profiler output files were downloaded and used as input for Cytoscape V3.7.

### Data plotting and statistical analysis

Geographical map was generated using R packages *tidyverse*, *maps*, and *mapplots*. Two-way *t* tests and Pearson association were performed using *Scipy* package in Python 3.7.

## Supplementary Information

**Additional file 1: Fig. S1.** Simplified model of the vitamin E biosynthesis pathway in barley. **Table S1.** 62 *HPT* homologous genes in 22 *Poales* species. **Table S2.** Amino acid sequence similarity comparison of HvHPT1 and HvHPT2 with other monocot HPTs. **Table S3.** Genotyping results of HPT2 in 113 bread wheat accessions. **Table S4.** The content of tocochromanol isomers in T1 leaves and T2 grains of transgenic lines. **Table S5.** Conserved elements in the promoter of *HvHPT1* and *HvHPT2*. **Table S6.** Primers for sequence amplification and genotyping.

**Additional file 2.** Sequence alignment and phylogenetic tree files for Fig. 1A, B, and Fig. 2F.

**Additional file 3 **Synteny alignment for *HPT1* and *HPT2* across 7 *Poales* species.

**Additional file 4.** SNP data for HPT2 in 300 barley pangenome lines and 109 Tibetan barley lines.

**Additional file 5.** PAML input and output files used for natural selection analysis in Table 1.

**Additional file 6.** Phylogeny-based orthologous gene inference across barley, wild emmer, and bread wheat and calculated gene retention rate for each orthologous gene group (hierarchical orthologous gene group at node N1 in Fig. 4A).

**Additional file 7.** Orthologous gene matrix (presence/absence) in barley, wild emmer, and bread wheat pangenome lines.

**Additional file 8.** List of the identified 326 HPT2-pattern-like genes (using barley as reference) and their corresponding gene retention rates in barley, wild emmer, and bread wheat.

**Additional file 9.** Gene duplication type data in Fig. 4C (326 HPT2-pattern-like genes) and Fig. 6B (three categories of genes).

**Additional file 10.** Lists of identified genes and gene annotations used for functional enrichment analyses.

**Additional file 11.** Ka, Ks, and Ka/Ks calculation results for HPT2-pattern-like genes and their parental genes in barley, wild emmer, and bread wheat.

**Additional file 12.** Sequence alignment, phylogenetic trees, and natural selection analyses data for Fig. 5E-O.

**Additional file 13.** Gene annotation validation results of selected OGs genes in 15 wheat genome assemblies.

**Additional file 14.** Original protoplast imaging files of barley.

## Data Availability

The datasets generated and/or analyzed during the current study are available at figshare repository: https://figshare.com/s/16286485ba63e682a400 [39–42]. The original input and output data files were deposited in GitHub public repository at https://github.com/yongjiam/HPT2_study [39–42].

## Abbreviations

EC: Evolution Canyon
GO: Gene Ontology
OG: Orthologous Group
GRR: Gene retention rate
*R*_*bw*_: GRR_barley / GRR_wheat
HOG: Hierarchical orthologous group
HGGT: Homogentisate geranylgeranyl transferase
HPT: Homogentisate phytyltransferase
LRTs: Likelihood ratio tests
ML: Maximum likelihood
NJ: Neighbor joining
Ka: Non-synonymous substitutions
NFS: North-facing slope
PAV: Presence/absence variation
RBGDs: Recent burst of gene duplications
SNP: Single-nucleotide polymorphism
SSDs: Small-scale duplications
SFS: South-facing slope
Ks: Synonymous substitutions
TPM: Transcripts per million reads
WGDs: Whole genome duplications
