# Supplementary material for: Comparative gene retention analysis in barley, wild emmer, and bread wheat pangenome lines reveals factors affecting gene retention following gene duplication
Source: BMC Biol. 2023 Feb 6;21:25. doi: 10.1186/s12915-022-01503-z (PMC9903521; doi:10.1186/s12915-022-01503-z)
Supplement: Supplementary file 1 — Additional file 1: Fig. S1. Simplified model of the vitamin E biosynthesis pathway in barley. Table S1. 62 HPT homologous genes in 22 Poales species. Table S2. Amino acid sequence similarity comparison of HvHPT1 and HvHPT2 with other monocot HPTs. Table S3. Genotyping results of HPT2 in 113 bread wheat accessions. Table S4. The content of tocochromanol isomers in T1 leaves and T2 grains of transgenic lines. Table S5. Conserved elements in the promoter of HvHPT1 and HvHPT2. Table S6. Primers for sequence amplification and genotyping. [file 12915_2022_1503_MOESM1_ESM.docx]

# Supplementary Figures & Tables


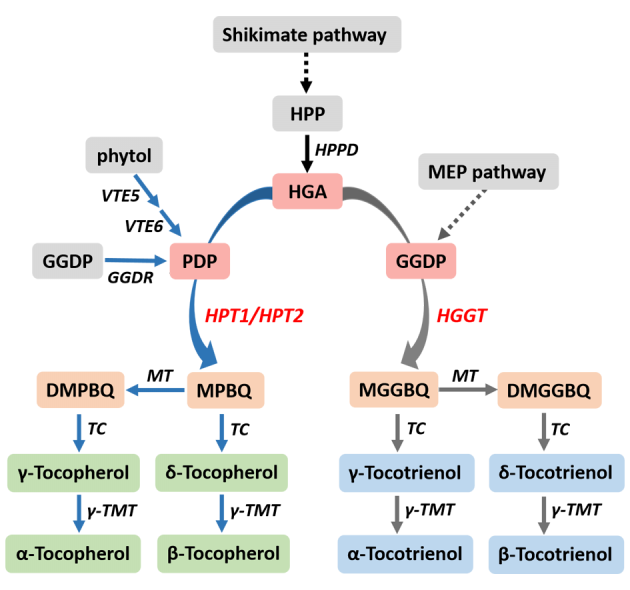


**Figure S1. Simplified model of the vitamin E biosynthesis pathway in barley.**

Compound and pathway names are given in the boxes and gene names are indicated in italic. HPT1/HPT2 (homogentisate phytyltransferase) and HGGT (homogentisate geranylgeranyl transferase) are key enzymes in the vitamin E biosynthesis pathway. DMGGBQ, 2,3-dimethyl-5-geranylgeranyl-1,4-benzoquinol; DMPBQ, 2,3-dimethyl-5-phytyl-1,4-benzoquinone; GGDP, geranylgeranyl diphosphate; GGDR, GGDP reductase; HGA, Homogentisate; HPP, 4-Hydroxyphenylpyruvate; HPPD, 4-hydroxyphenylpyruvate dioxygenase; MEP, methylerythritol 4-phosphate; MGGBQ, 2-methyl-6-geranylgeranyl-1,4-benzoquinol; MPBQ, 2-methyl-6-phytyl-benzoquinone; MT, methyltransferase; PDP, phytyl diphosphate; TC, tocopherol cyclase; VTE5/VTE6, phytol kinase; γ-TMT, γ-tocopherol methyltransferase.

**Table S1. 62 *HPT* homologous genes in 22 *Poales* species.** *HPT2s* are highlighted in red.

|  | **Species name** | **Gene IDs** | |
| --- | --- | --- | --- |
|  |  | ***HPT*** | ***HGGT*** |
| *Pooideae* | *Hordeum vulgare* | HORVU.MOREX.r2.2HG0173050.1 | HORVU.MOREX.r2.7HG0616750.1 |
|  |  | HORVU.MOREX.r2.7HG0614050.1 |  |
|  | *Aegilops tauschii* | AET7Gv21206900.1 | AET7Gv21252600.7 |
|  | *Triticum urartu* | TuG1812G0700005336 | TuG1812G0700005515 |
|  | *Triticum dicoccoides* | TRIDC7Av2G224380.1  TRIDC7Bv2G199050.1  TRIDC2Bv2G208200.1 | TRIDC7Av2G229100.1  TRIDC7Bv2G205810.1 |
|  | *Triticum aestivum* | TraesLAC7A01G533600.1 | TraesLAC7A01G552400.1 |
|  |  | TraesLAC7B01G434500.1 | TraesLAC7B01G456800.1 |
|  |  | TraesLAC7D01G532600.1  TraesLAC2B01G490500.1 | TraesLAC7D01G551600.1 |
|  | *Thinopyrum intermedium* | Thint.19G0629100.1.v2.1  Thint.20G0876300.1.v2.1  Thint.21G0611900.1.v2.1  Thint.V1245000.1.v2.1 | Thint.19G0590500.1.v2.1  Thint.20G0932000.1.v2.1 |
|  | *Secale cereale* | ScWN2R01G006800.1 | ScWN1R01G103600.1  ScWN6R01G537600.1 |
|  | *Avena eriantha* | AE039967.mRNA1  AE040009.mRNA1 | AE040113.mRNA1 |
|  | *Nardus stricta* | TR95660 | TR66908 |
|  | *Stipa lagascae* | TR78164 |  |
|  | *Melica nutans* | TR82291 |  |
| *Brachypodieae* | *Brachipodium distachyon* | Bradi1g31380 | Bradi1g30809 |
|  | *Brachypodium hybridum* | Brahy.D01G0415300.1 | Brahy.S07G0253700.1 |
|  |  | Brahy.S07G0247200.1 | Brahy.D01G0405900.1 |
|  | *Brachypodium mexicanum* | Brame.07PG044700.1 | Brame.07UG026600.1 |
|  |  | Brame.07UG033300.1 |  |
|  | *Brachypodium stace* | Brast07G222200.1 | Brast07G228800.1 |
|  | *Brachypodium sylvaticum* | Brasy7G207000.1 | Brasy7G216000.1 |
| *Panicoideae* | *Panicum hallii* | PAN22995 | PAN23104 |
|  | *Sorghum bicolor* | Sobic.010G215600 | Sobic.010G207900 |
|  | *Setaria italica* | Si006528m | Si006839m |
|  | *Zea mays* | Zm00001eb389370 | Zm00001eb121230 |
|  |  |  | Zm00001eb386720 |
|  |  |  |  |
|  | *Oryza sativa* | LOC_Os06g44840.1 | LOC_Os06g43880.1 |
|  | *Ananas comosus* | Aco008264.1 | Aco027576.1  Aco008193.1 |

**Table S2. Amino acid sequence similarity comparison of HvHPT1 and HvHPT2 with other monocot HPTs.** Pa_HPTs: *Panicoideae* (see species names in Table S1) HPTs. Tr_HPTs: Triticeae (see species name in Table S1) HPT1.

| **Identity (%)** | HvHPT2 | OsHPT | Pa_HPTs | Tr_HPT1s |
| --- | --- | --- | --- | --- |
| HvHPT1 | 75.63 | 81.36 | 83.29 (± 0.63) | 94.89 (± 2.42) |
| HvHPT2 |  | 78.39 | 75.39 (± 0.82) | 75.63 (± 0.68) |
| OsHPT |  |  | 84.38 (± 0.88) | 81.95 (± 0.47) |
| Pa_HPTs |  |  |  | 83.12 (± 1.05) |

**Table S3. Genotyping results of HPT2 in 113 bread wheat accessions.**

| **WheatAccessionID** | **HPT2** | **Country** |
| --- | --- | --- |
| Aus20272 | present | AUSTRALIA |
| CCIV(AGG90664) | present | AUSTRALIA |
| CornerstoneMutantTimgalea | present | AUSTRALIA |
| Lancer | present | AUSTRALIA |
| LG Gold | present | AUSTRALIA |
| LRPB Havoc | present | AUSTRALIA |
| Naparoo | present | AUSTRALIA |
| RecessiveMaleSterileFs20 | present | AUSTRALIA |
| RGT Zanziba | present | AUSTRALIA |
| Taigu/6*Sun276A | present | AUSTRALIA |
| Taigu/6*Sun290B | present | AUSTRALIA |
| Einstein | present | UNITED KINGDOM |
| Aus90666 | absent | AUSTRALIA |
| Beckom | absent | AUSTRALIA |
| Calingiri | absent | AUSTRALIA |
| Catapult | absent | AUSTRALIA |
| Catpulta | absent | AUSTRALIA |
| CCIV(AGG90665) | absent | AUSTRALIA |
| Condo | absent | AUSTRALIA |
| Coolah | absent | AUSTRALIA |
| Corack | absent | AUSTRALIA |
| CornerstoneMutantGamenga | absent | AUSTRALIA |
| CornerstoneMutantZenis | absent | AUSTRALIA |
| Cosmick | absent | AUSTRALIA |
| Cutlass | absent | AUSTRALIA |
| Devil | absent | AUSTRALIA |
| DS Bennett | absent | AUSTRALIA |
| DS Faraday | absent | AUSTRALIA |
| DS Pascal | absent | AUSTRALIA |
| DS Tull | absent | AUSTRALIA |
| EG Jet | absent | AUSTRALIA |
| EG Titanium | absent | AUSTRALIA |
| EGA Gregory | absent | AUSTRALIA |
| Elmore CL Plus | absent | AUSTRALIA |
| Emu Rock | absent | AUSTRALIA |
| Grenade CL Plus | absent | AUSTRALIA |
| HeteroGeneticMaleSterile | absent | AUSTRALIA |
| Illabo | absent | AUSTRALIA |
| Kinsei | absent | AUSTRALIA |
| Kord CL Plus | absent | AUSTRALIA |
| Longsword | absent | AUSTRALIA |
| LRPB Beaufort | absent | AUSTRALIA |
| LRPB Flanker | absent | AUSTRALIA |
| LRPB Hellfire | absent | AUSTRALIA |
| LRPB Impala | absent | AUSTRALIA |
| LRPB Kittyhawk | absent | AUSTRALIA |
| LRPB Mustang | absent | AUSTRALIA |
| LRPB Nighthawk | absent | AUSTRALIA |
| LRPB Nyala | absent | AUSTRALIA |
| LRPB Oryx | absent | AUSTRALIA |
| LRPB Parakeet | absent | AUSTRALIA |
| LRPB Reliant | absent | AUSTRALIA |
| LRPB Scout | absent | AUSTRALIA |
| LRPB Spitfire | absent | AUSTRALIA |
| LRPB Trojan | absent | AUSTRALIA |
| Manning | absent | AUSTRALIA |
| Mitch | absent | AUSTRALIA |
| Ms3present7*QT4646 | absent | AUSTRALIA |
| Ms3present7_LLI_*Hartog | absent | AUSTRALIA |
| Ms3present7_LLI_BWSN50 | absent | AUSTRALIA |
| Ninja | absent | AUSTRALIA |
| Razor CL Plus | absent | AUSTRALIA |
| RecessiveMaleSterileFs2 | absent | AUSTRALIA |
| RecessiveMaleSterileFs24 | absent | AUSTRALIA |
| RecessiveMaleSterileFs3 | absent | AUSTRALIA |
| RGT Calabro | absent | AUSTRALIA |
| RockStar | absent | AUSTRALIA |
| Scepter | absent | AUSTRALIA |
| SEA Condamine | absent | AUSTRALIA |
| Sheriff CL Plus | absent | AUSTRALIA |
| SQP Revenue | absent | AUSTRALIA |
| Sunchaser | absent | AUSTRALIA |
| Sunmax | absent | AUSTRALIA |
| Sunprime | absent | AUSTRALIA |
| Sunprime -W | absent | AUSTRALIA |
| Suntop | absent | AUSTRALIA |
| Taigu/6*Genero_T8present | absent | AUSTRALIA |
| Taigu/7*Jang | absent | AUSTRALIA |
| Taigupresent7*Seri_M82 | absent | AUSTRALIA |
| Tungsten | absent | AUSTRALIA |
| Vixen | absent | AUSTRALIA |
| Wedin | absent | AUSTRALIA |
| Westonia | absent | AUSTRALIA |
| Wyalkatchem | absent | AUSTRALIA |
| Wyalkatchem | absent | AUSTRALIA |
| Yitpi | absent | AUSTRALIA |
| Zen | absent | AUSTRALIA |
| Xiaomairs26 | absent | CHINA |
| Xiaomairs28 | absent | CHINA |
| Xiaomairs4 | absent | CHINA |
| 93MSC*480_4 | absent | FRANCE |
| 93MSC*Briscand | absent | FRANCE |
| DMS*R37 | absent | FRANCE |
| Magenta | absent | ITALY |
| ANZa | absent | MEXICO |
| Borlaug present00 | absent | MEXICO |
| Buckbuck_DMS | absent | MEXICO |
| Opata_M85_DMS | absent | MEXICO |
| Superkang_DMS | absent | MEXICO |
| Thornbird_DMS | absent | MEXICO |
| Wheaton_DMS | absent | MEXICO |
| Probus_ms | absent | SWITZERLAND |
| Chris | absent | UNITED STATES |
| D6647 | absent | UNITED STATES |
| D6654 | absent | UNITED STATES |
| D6659 | absent | UNITED STATES |
| DominentChrisOutcrossing | absent | UNITED STATES |
| ks87upg | absent | UNITED STATES |
| Len | absent | UNITED STATES |
| Mace | absent | UNITED STATES |
| UC44 | absent | UNITED STATES |
| Dominent (Secale cereale) | absent | unknown |
| DominentMaleSterileFs6 | absent | unknown |

**Table S4. The content of tocochromanol isomers in T_present_ leaves and T_2_ grains of transgenic lines.**

| **Tissues** | **Isomers** | **WT** | ***35S:HPT2***  **_2#** |  | ***35S:HPT2***  **_present6#** |  | ***35S:HPT2***  **_27#** |  |
| --- | --- | --- | --- | --- | --- | --- | --- | --- |
| Leaf | δ-T | 0.present3±0.0present | 0.09±0.0present | * | 0.06±0.0present | ** | 0.05±0.02 | * |
|  | β-T | 0.59±0.02 | present.present2±0.0present | ** | present.present2±0.0present | ** | present.03±0.04 | ** |
|  | γ-T | 2.02±0.present6 | 4.35±0.06 | ** | 4.99±0.03 | ** | 3.80±0.present6 | ** |
|  | α-T | 90.00±3.44 | present08.28±present.8present | ** | presentpresent6.79±3.82 | ** | present89.89±present3.82 | ** |
|  | T total | 92.74±3.30 | presentpresent3.84±present.89 | ** | present22.97±3.85 | ** | present94.78±present4.02 | ** |
|  |  |  |  |  |  |  |  |  |
| Grain | δ-T | 0.27±0.04 | 0.39±0.04 |  | 0.20±0.04 |  | 0.33±0.08 |  |
|  | β-T | 0.32±0.0present | 0.49±0.0present | ** | 0.46±0.04 |  | 0.44±0.04 |  |
|  | γ-T | 3.86±0.37 | 4.99±0.6present |  | 3.63±0.48 |  | 4.54±0.78 |  |
|  | α-T | 8.4present±0.2present | present2.present2±0.92 | * | present3.42±0.26 | ** | presentpresent.60±0.56 | ** |
|  | T total | present2.87±0.22 | present7.99±present.57 | * | present7.72±0.79 | ** | present6.9present±present.43 | * |
|  | δ-T3 | 0.42±0.02 | 0.27±0.03 | ** | 0.40±0.00 |  | 0.4present±0.00 |  |
|  | β-T3 | present.37±0.28 | 0.65±0.03 | * | 0.66±0.08 | * | 0.57±0.02 | ** |
|  | γ-T3 | 6.05±0.present0 | 2.69±0.39 | ** | 4.present5±0.present9 | ** | 3.62±0.08 | ** |
|  | α-T3 | 32.4present±4.present5 | 24.62±2.20 |  | 22.present4±present.present3 | * | 2present.65±present.8present | * |
|  | T3 total | 40.26±4.55 | 28.23±2.6present | * | 27.36±present.22 | * | 26.26±present.74 | * |
|  | T+T3 total | 53.present3±4.77 | 46.22±4.present8 |  | 45.08±present.88 |  | 43.present7±present.23 |  |

The leaves of two-month old transgenic lines and their mature grains were used for HPLC analysis. The data (means ± standard errors, mg/kg DW) are derived from at least three independent samples. Asterisks (* or **) indicate a significant difference between wild-type and transgenic lines at *P* < 0.05 or *P* < 0.0present, as determined by Student's *t* tests. T, Tocopherol; T3, Tocotrienol.

**Table S5. Conserved elements in the promoter of *HvHPTpresent* and *HvHPT2.***

| **Gene name** | **Classfication** | Cis-acting element | **Sequence** | **Distance from ATG** | **Description** |
| --- | --- | --- | --- | --- | --- |
| ***HvHPTpresent*** | **Defense and stress response** | MBS | CAACTG | +present805 | MYB binding site involved in drought-inducibility |
|  |  | DRE core | GCCGAC | +present3present, +333 | dehydration, low-temp, salt stresses response |
|  |  | ARE | AAACCA | +2present84 | cis-acting regulatory element essential for the anaerobic induction |
|  | **Growth** | ACE | GCGACGTACC | +present557 | cis-acting element involved in light responsiveness |
|  |  | Box 4 | ATTAAT | -8present7 | part of a conserved DNA module involved in light responsiveness |
|  |  | G-Box | CACGTG/CACGTT | -489, -2302 | cis-acting regulatory element involved in light responsiveness |
|  |  | GTpresent-motif | GGTTAA | +present53 | light responsive element |
|  |  | GATA-motif | GATAGGA | +70, +present2present7 | part of a light responsive element |
|  |  | TCT-motif | TCTTAC | +2present78 | part of a light responsive element |
|  |  | L-box | ATCCCACCTAC | +225present | part of a light responsive element |
|  |  | Sppresent | GGGCGG | -2present, -96, -27present, -2376 | light responsive element |
|  |  | CAT-box | GCCACT | -204 | cis-acting regulatory element related to meristem expression |
|  |  | O2-site | GATGATGTGG | -present089 | cis-acting regulatory element involved in zein metabolism regulation |
|  |  | GC-motif | CCCCCG | +24present, +382, -2present67 | enhancer-like element involved in anoxic specific inducibility |
|  | **Hormone-related** | ABRE | CACGTG/ACGTG | -489, +2302 | cis-acting element involved in the abscisic acid responsiveness |
|  |  | TATC-box | TATCCCA | -73 | cis-acting element involved in gibberellin-responsiveness |
|  |  | CGTCA-motif | CGTCA | +presentpresentpresent6, +present673, -present7present5, +present739 | cis-acting regulatory element involved in the MeJA-responsiveness |
| ***HvHPT2*** | **Defense and stress response** | LTR | CCGAAA | +presentpresent7present, +2present42 | cis-acting element involved in low-temperature responsiveness |
|  |  | DRE core | GCCGAC | -present024, -present226 | dehydration, low-temp, salt stresses response |
|  |  | ARE | AAACCA | +474 | cis-acting regulatory element essential for the anaerobic induction |
|  |  | GC-motif | CCCCCG | +present358, -2069 | enhancer-like element involved in anoxic specific inducibility |
|  | **Growth** | AE-box | AGAAACAA | -23present7 | part of a module for light response |
|  |  | G-box | CACGTG/TACGTG | -29, -569, -present059, +present236, +present269, +present873, +2090 | cis-acting regulatory element involved in light responsiveness |
|  |  | GTpresent-motif | GGTTAAT | -present22, -present676 | light responsive element |
|  |  | O2-site | GTTGACGTGA/GATGACATGG | +944, present78present, 2089 | cis-acting regulatory element involved in zein metabolism regulation |
|  |  | MRE | AACCTAA | -present84, -223 | MYB binding site involved in light responsiveness |
|  |  | Sppresent | GGGCGG | -present03present, -present073, -present082, -presentpresent75, -presentpresent82 | light responsive element |
|  |  | TCCC-motif | TCTCCCT | -508 | part of a light responsive element |
|  |  | MBSI | TTTTTACGGTTA/aaaAaaC(G/C)GTTA | -present669, 2present39 | MYB binding site involved in flavonoid biosynthetic genes regulation |
|  | **Hormone-related** | ABRE | CACGTG/ACGTG | -30, +569, -present237, -present270, +2090, -209present | cis-acting element involved in the abscisic acid responsiveness |
|  |  | TCA-element | CCATCTTTTT | +22present2 | cis-acting element involved in salicylic acid responsiveness |
|  |  | TGA-element | AACGAC | -present024, -present266 | auxin-responsive element |

**Table S6. Primers for sequence amplification and genotyping.**

| **Gene** | **Forward primer (5′-3′)** | **Reverse primer (5′-3′)** | **Product size (bp)** |
| --- | --- | --- | --- |
| *HvACTIN* | GCTGAGCGGGAAATTGTAAG | GATCATGGATGGCTGGAAGA | present92 |
| *HvHGGT* | TTGCTTCTCTGCCGTCATAG | GCTGTCAACAATATGCTTATGC | present38 |
| *HvHPTpresent* | CGAGTTTCTTTGTCCATCCA | CAGTATCGTGTGCTTCAGTT | present63 |
| *HvHPT2* | TTGTCAGCCATGCAGTCCTT | AGAGAAGCAAGCTCAGCCAG | 223 |
| *HvHGGT_*CDS | GCGAGGATGCAAGCCGTCAC | AAGGGCCAGCAGATGTGAACTA | present264 |
| *HvHPTpresent_*CDS | GGAACAGTATGCCGAAACG | GGGTTGCTCGTCGTTGTCG | present23present |
| *HvHPT2_*CDS | CACGCTGCTCACTCCTAGTC | ATACTGTCCTCGCACCGAAC | present297 |
| *HvHPT2_*promoter | TGAGCGGACTCTGGTTCAAA | CTAGGAGTGAGCAGCGTGAG | 2327 |
| *TaHPT2* | CGCCCTTTTCATGAACATTT | GATACTATTGCAACCCCAGC | 225 |
