# Supplementary figures and images for: Comparative gene retention analysis in barley, wild emmer, and bread wheat pangenome lines reveals factors affecting gene retention following gene duplication
Source: BMC Biol. 2023 Feb 6;21:25. doi: 10.1186/s12915-022-01503-z (PMC9903521; doi:10.1186/s12915-022-01503-z)

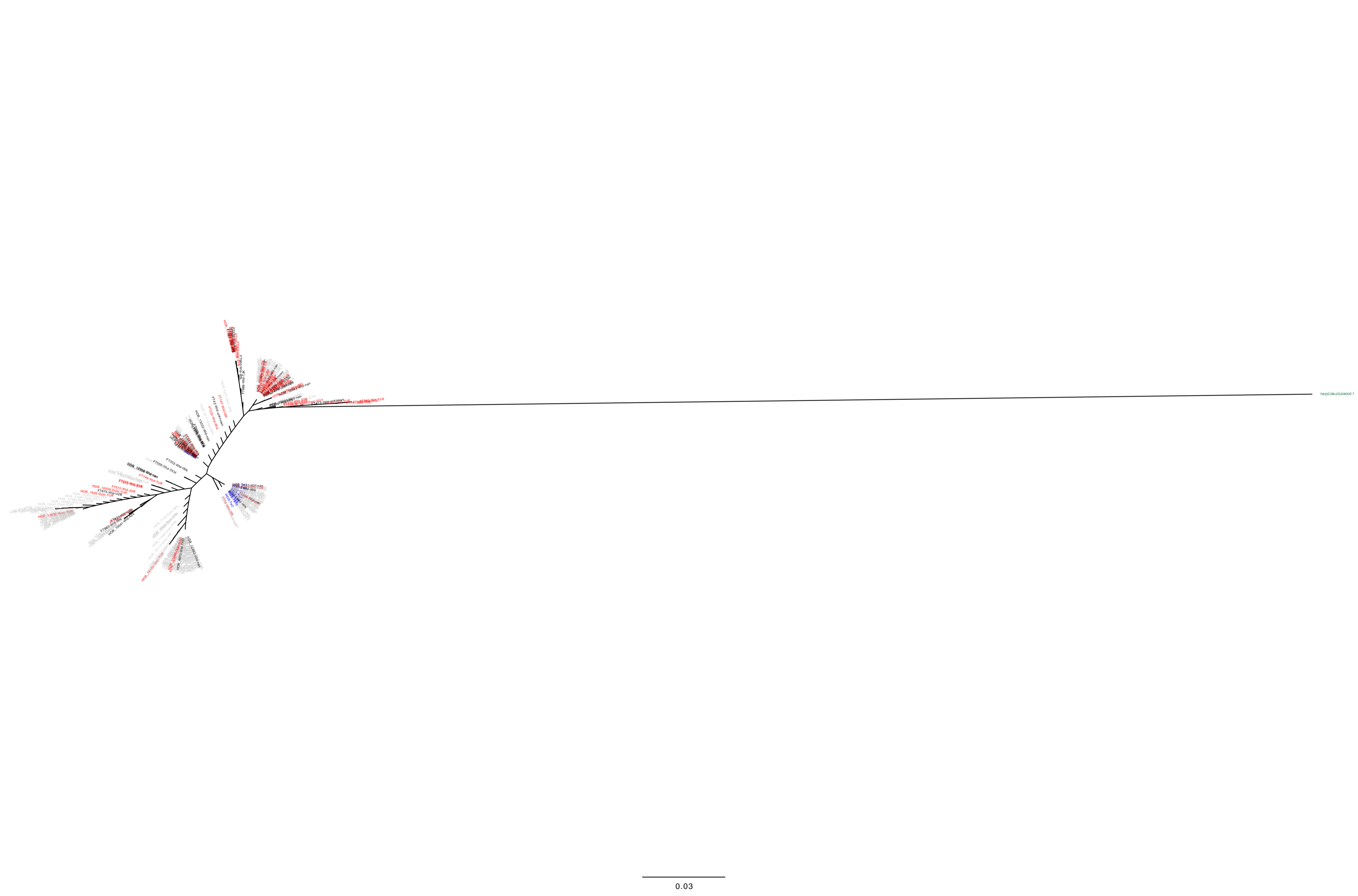

Supplement: Supplementary file 2 — Additional file 2. Sequence alignment and phylogenetic tree files for Fig. 1A, B, and Fig. 2F. [file 12915_2022_1503_MOESM2_ESM.zip › Files_S1/Figure2F_tree/Barley_pan_10Tiebetan_emmer_HPT2_CDS.treefile.pdf]

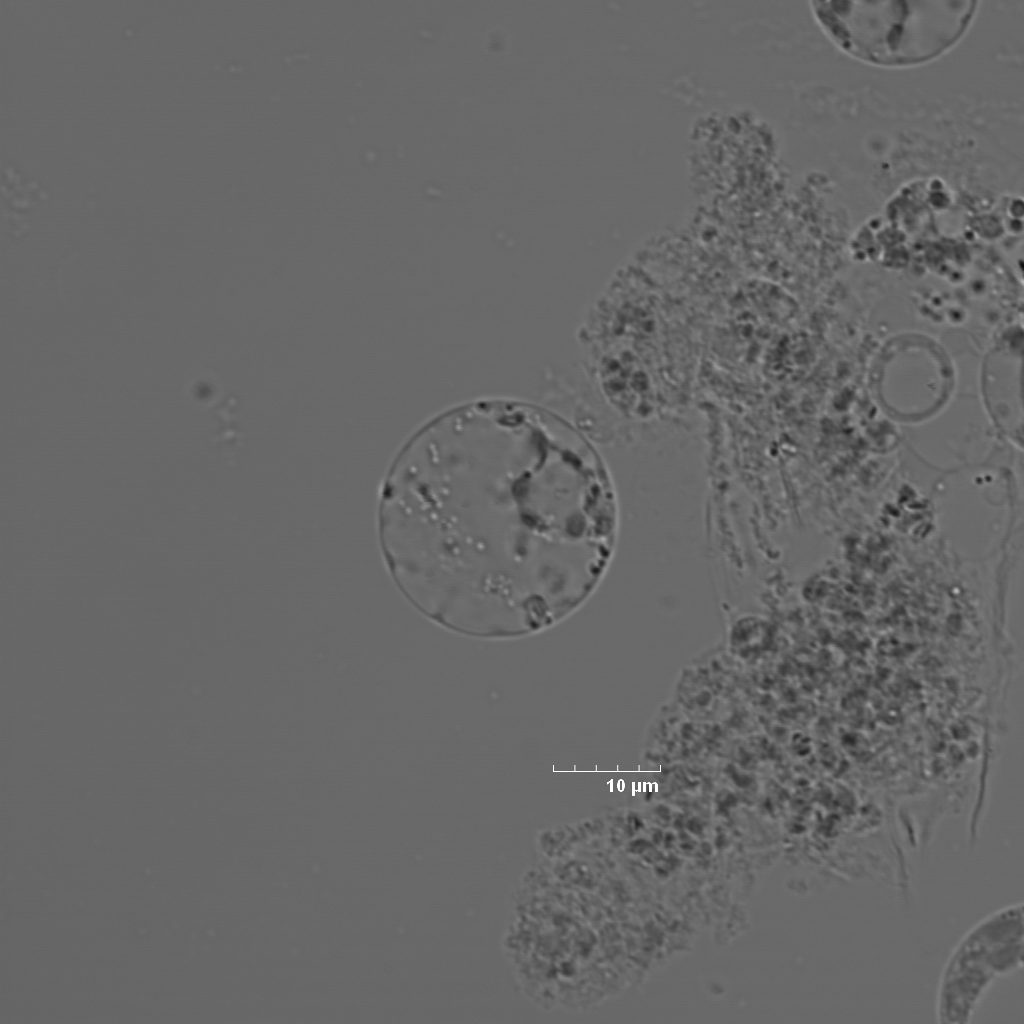

Supplement: Supplementary file 14 — Additional file 14. Original protoplast imaging files of barley. [file 12915_2022_1503_MOESM14_ESM.zip › Localization of protoplast in barley/control GFP/GFP_bright field.tif]

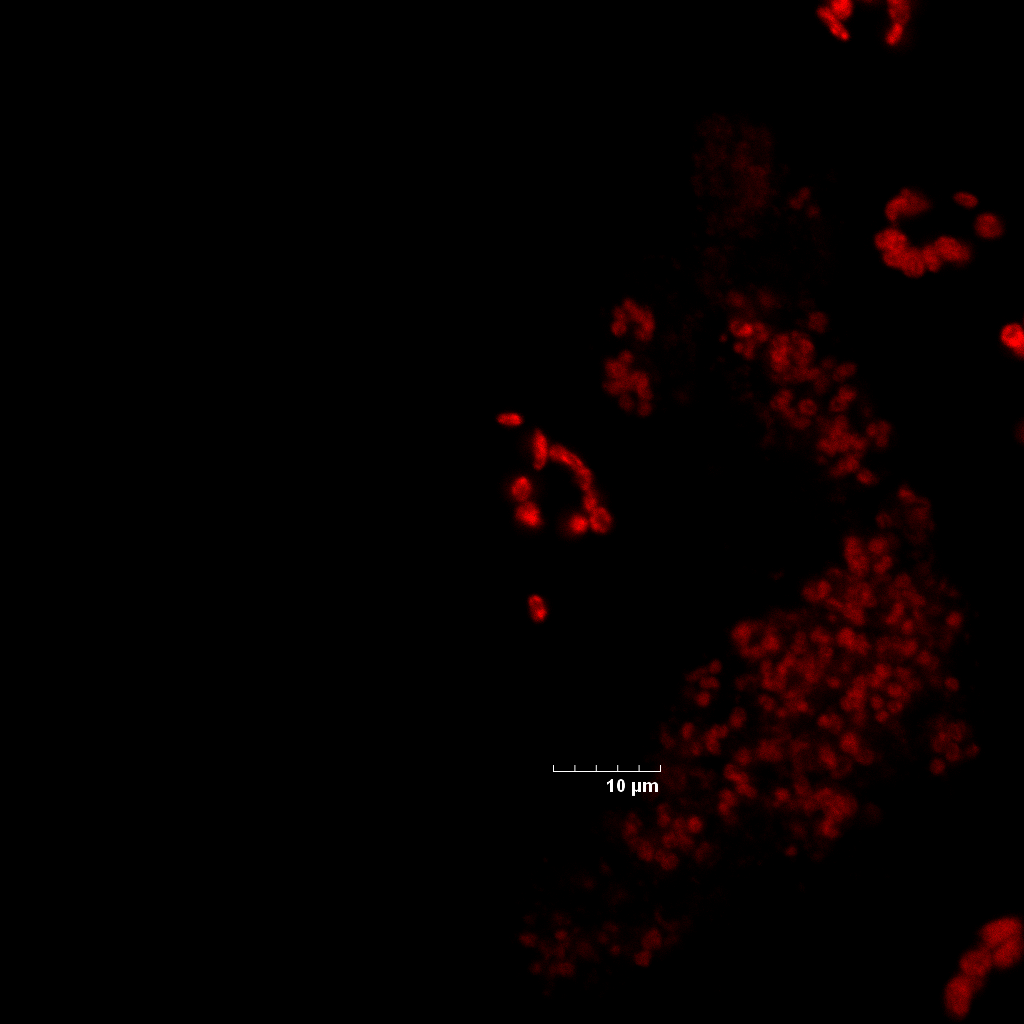

Supplement: Supplementary file 14 — Additional file 14. Original protoplast imaging files of barley. [file 12915_2022_1503_MOESM14_ESM.zip › Localization of protoplast in barley/control GFP/GFP_Chlorophyll.tif]

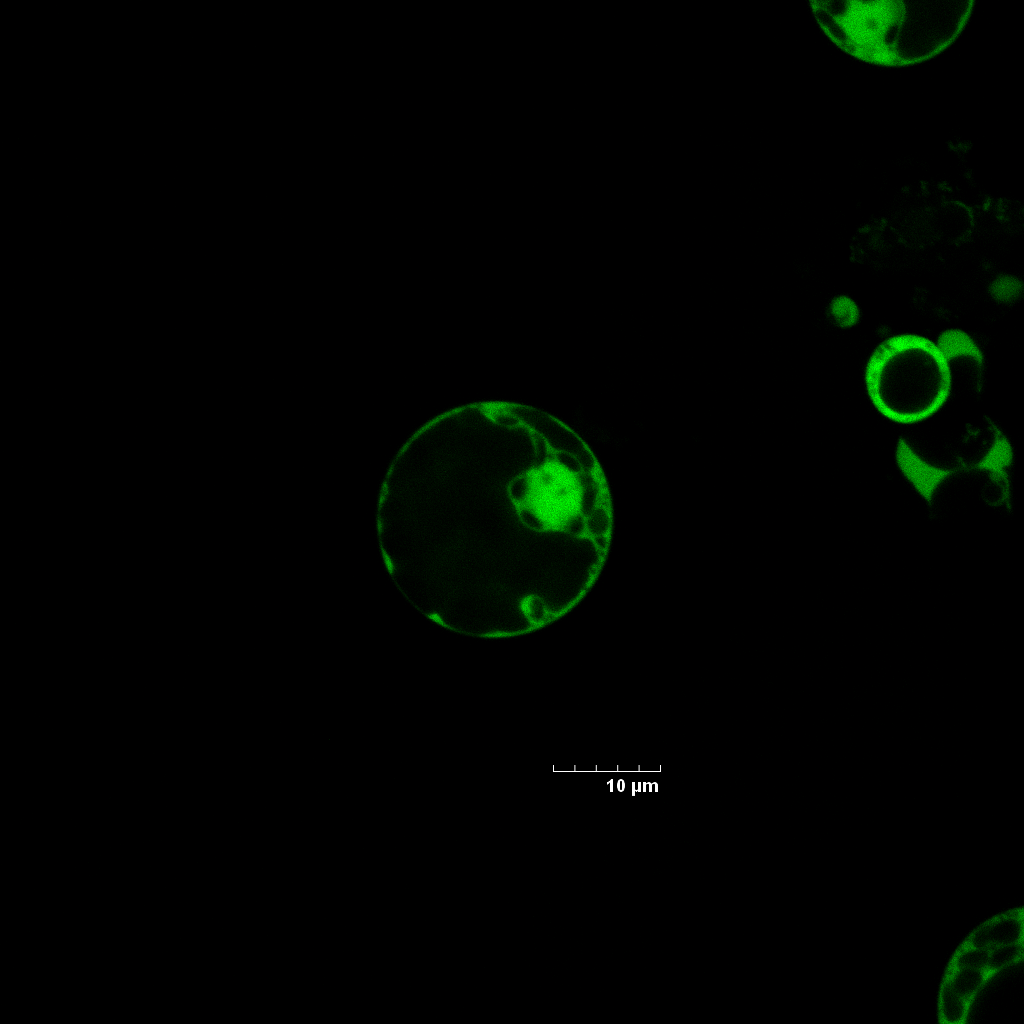

Supplement: Supplementary file 14 — Additional file 14. Original protoplast imaging files of barley. [file 12915_2022_1503_MOESM14_ESM.zip › Localization of protoplast in barley/control GFP/GFP_GFP.tif]

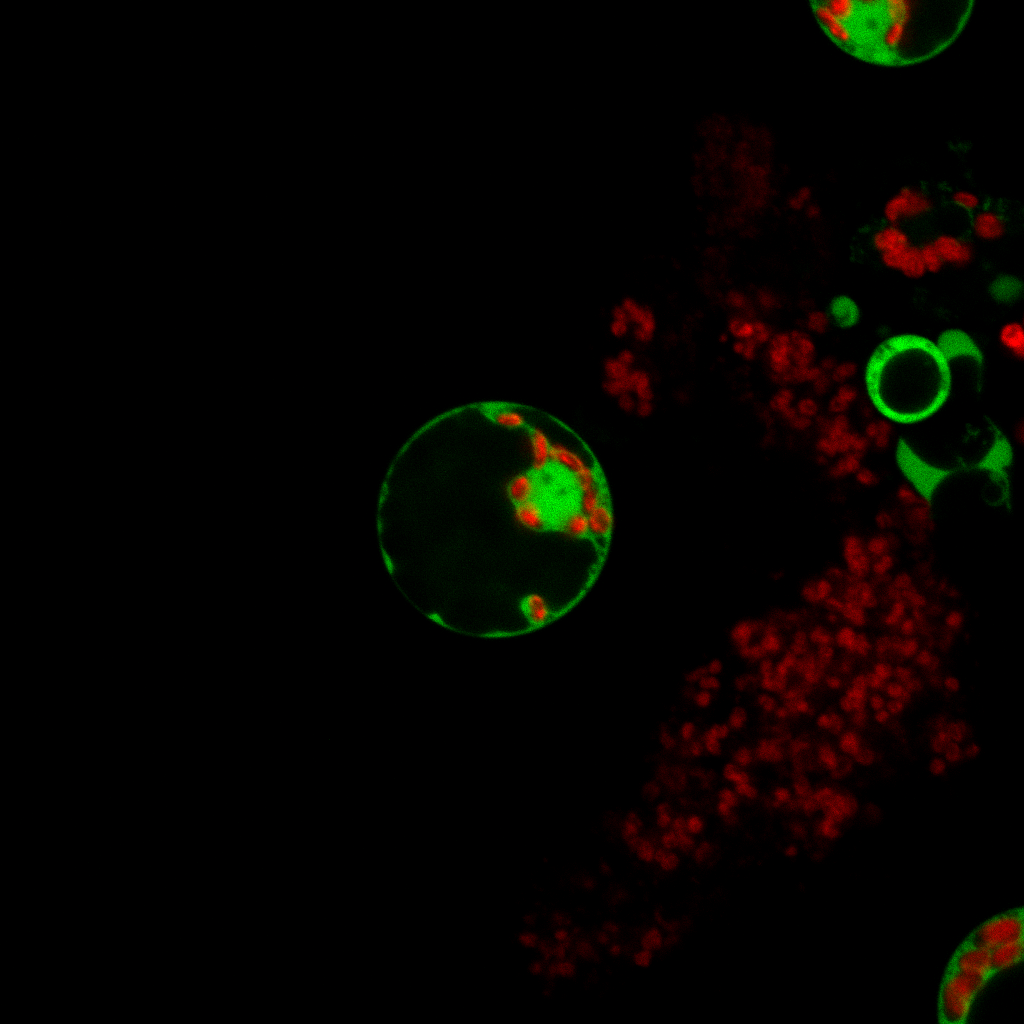

Supplement: Supplementary file 14 — Additional file 14. Original protoplast imaging files of barley. [file 12915_2022_1503_MOESM14_ESM.zip › Localization of protoplast in barley/control GFP/GFP_merged_1.tif]

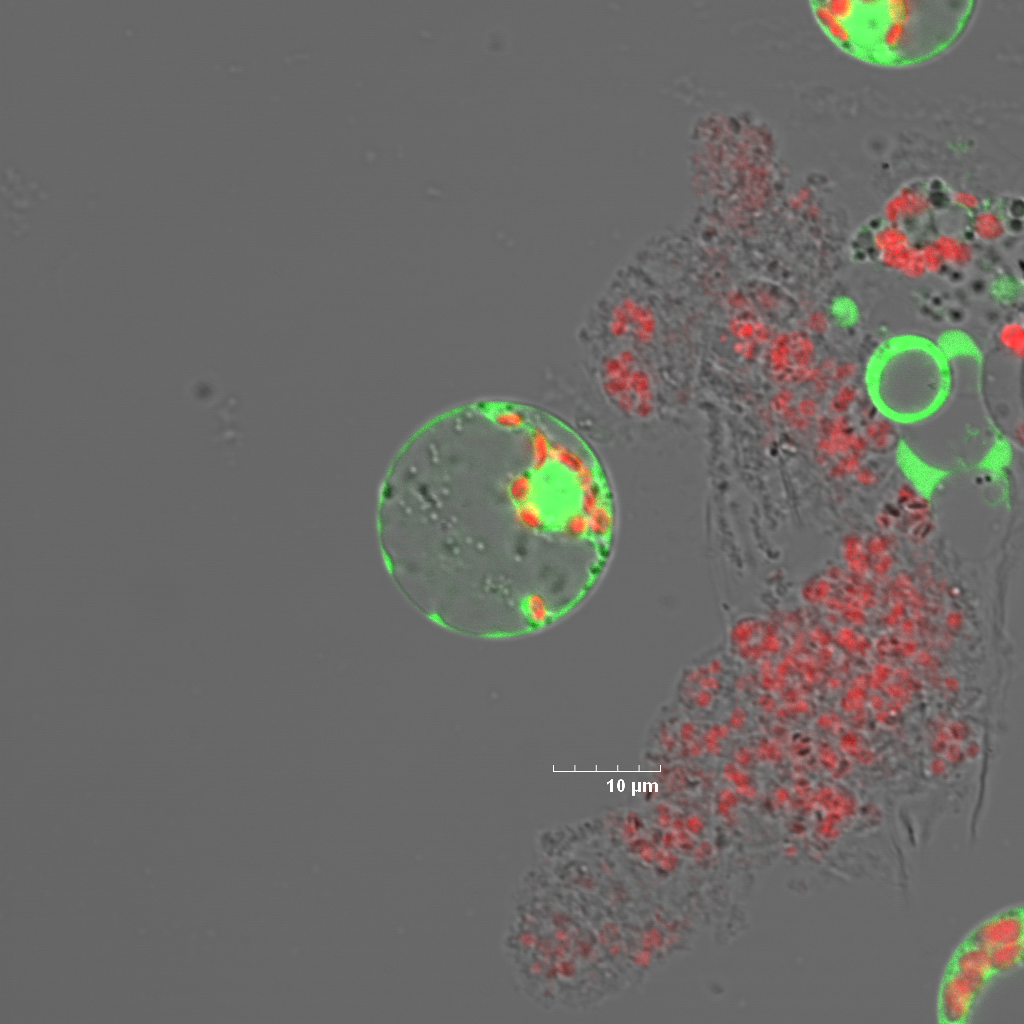

Supplement: Supplementary file 14 — Additional file 14. Original protoplast imaging files of barley. [file 12915_2022_1503_MOESM14_ESM.zip › Localization of protoplast in barley/control GFP/GFP_merged_2.tif]

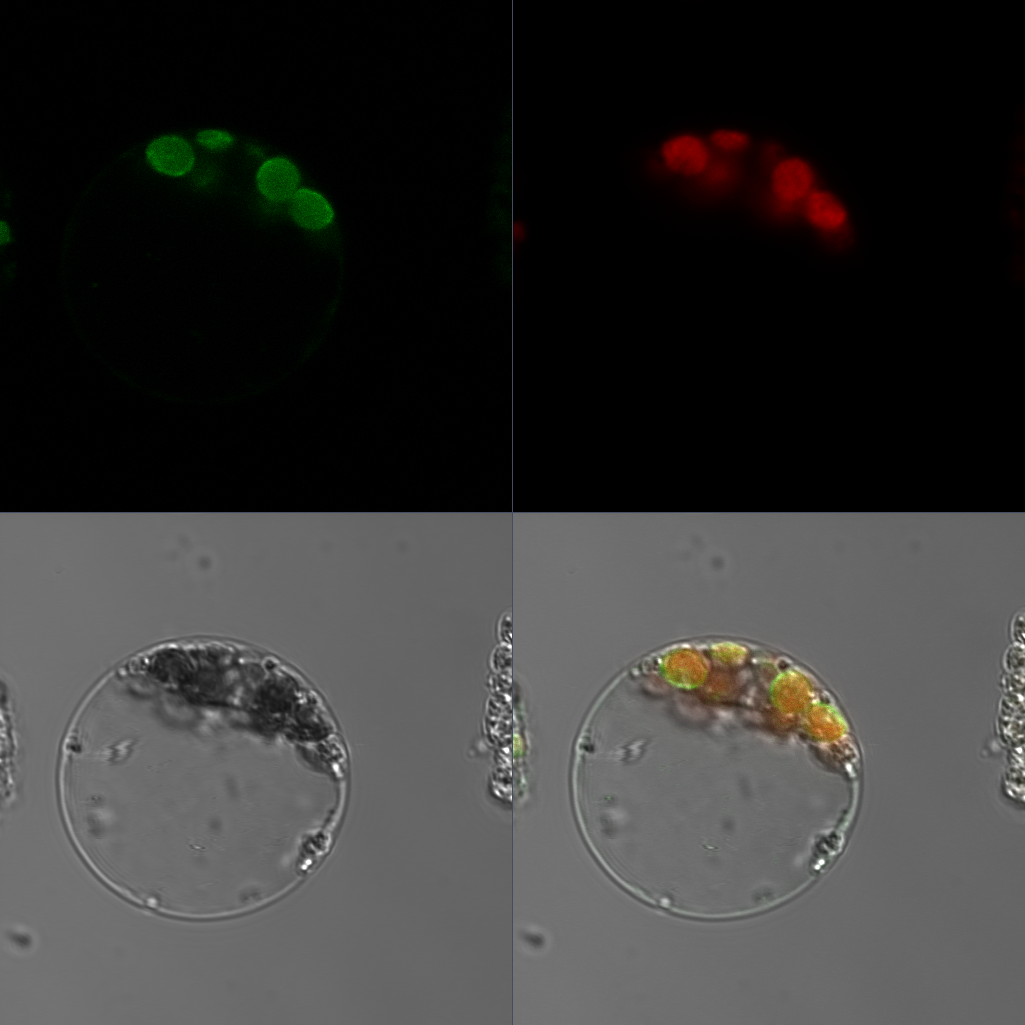

Supplement: Supplementary file 14 — Additional file 14. Original protoplast imaging files of barley. [file 12915_2022_1503_MOESM14_ESM.zip › Localization of protoplast in barley/HvHGGT_W5.tif]

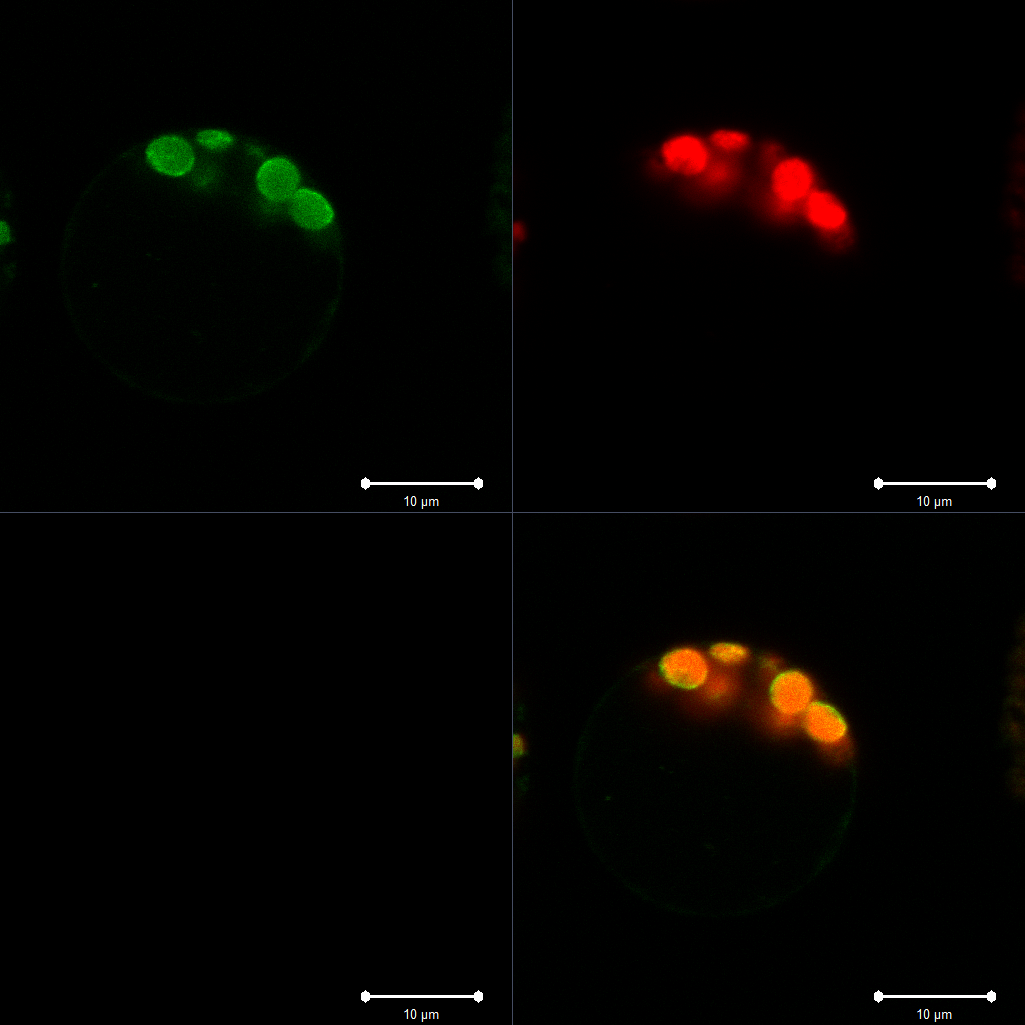

Supplement: Supplementary file 14 — Additional file 14. Original protoplast imaging files of barley. [file 12915_2022_1503_MOESM14_ESM.zip › Localization of protoplast in barley/HvHGGT_W5-max 10um.tif]

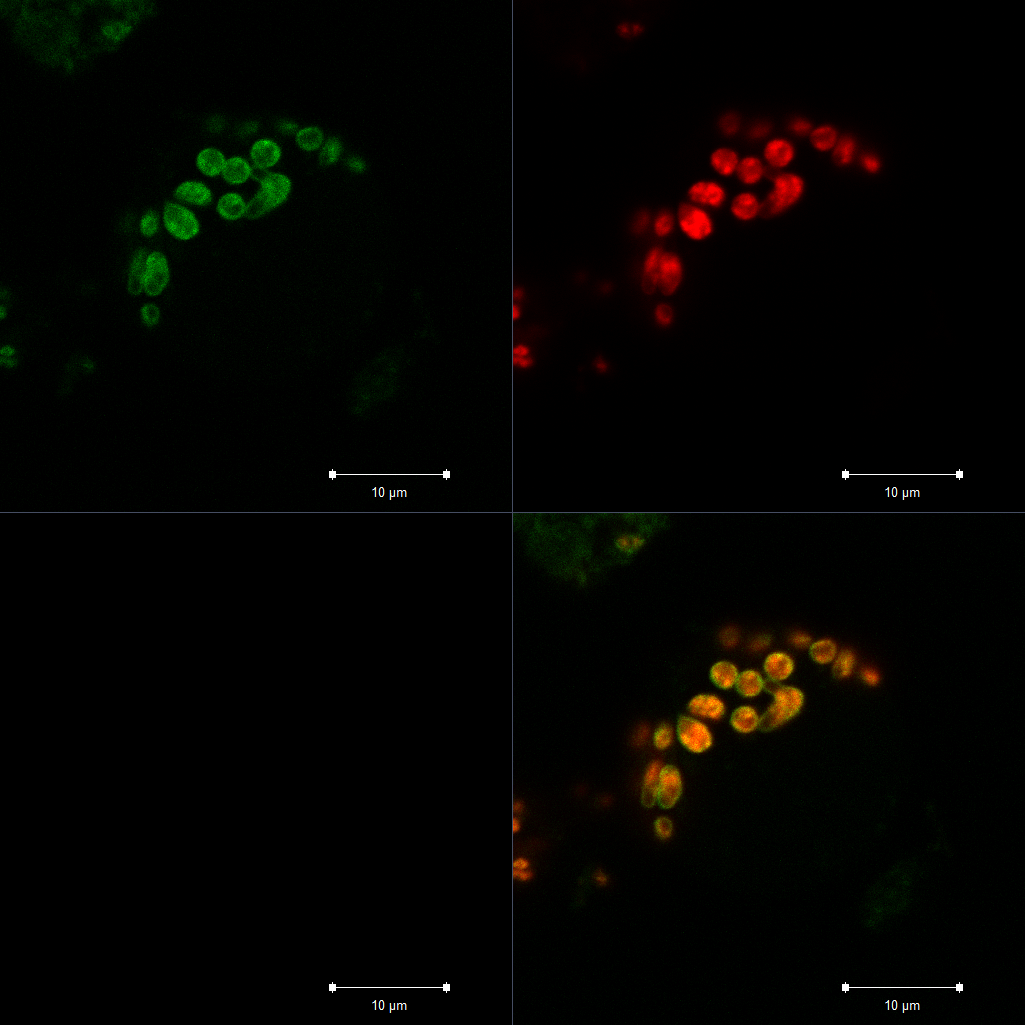

Supplement: Supplementary file 14 — Additional file 14. Original protoplast imaging files of barley. [file 12915_2022_1503_MOESM14_ESM.zip › Localization of protoplast in barley/HvHPT1_397W5-5 max.tif]

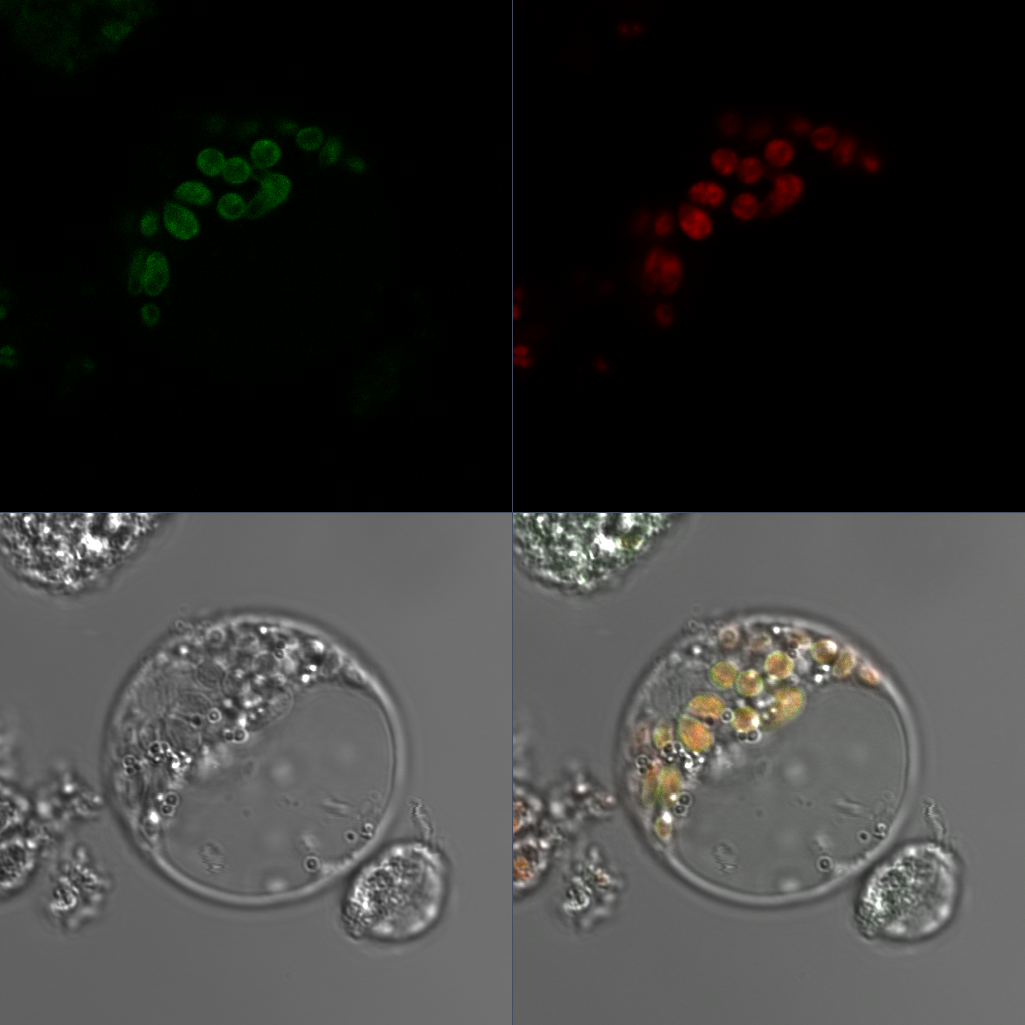

Supplement: Supplementary file 14 — Additional file 14. Original protoplast imaging files of barley. [file 12915_2022_1503_MOESM14_ESM.zip › Localization of protoplast in barley/HvHPT1_397W5-5.tif]

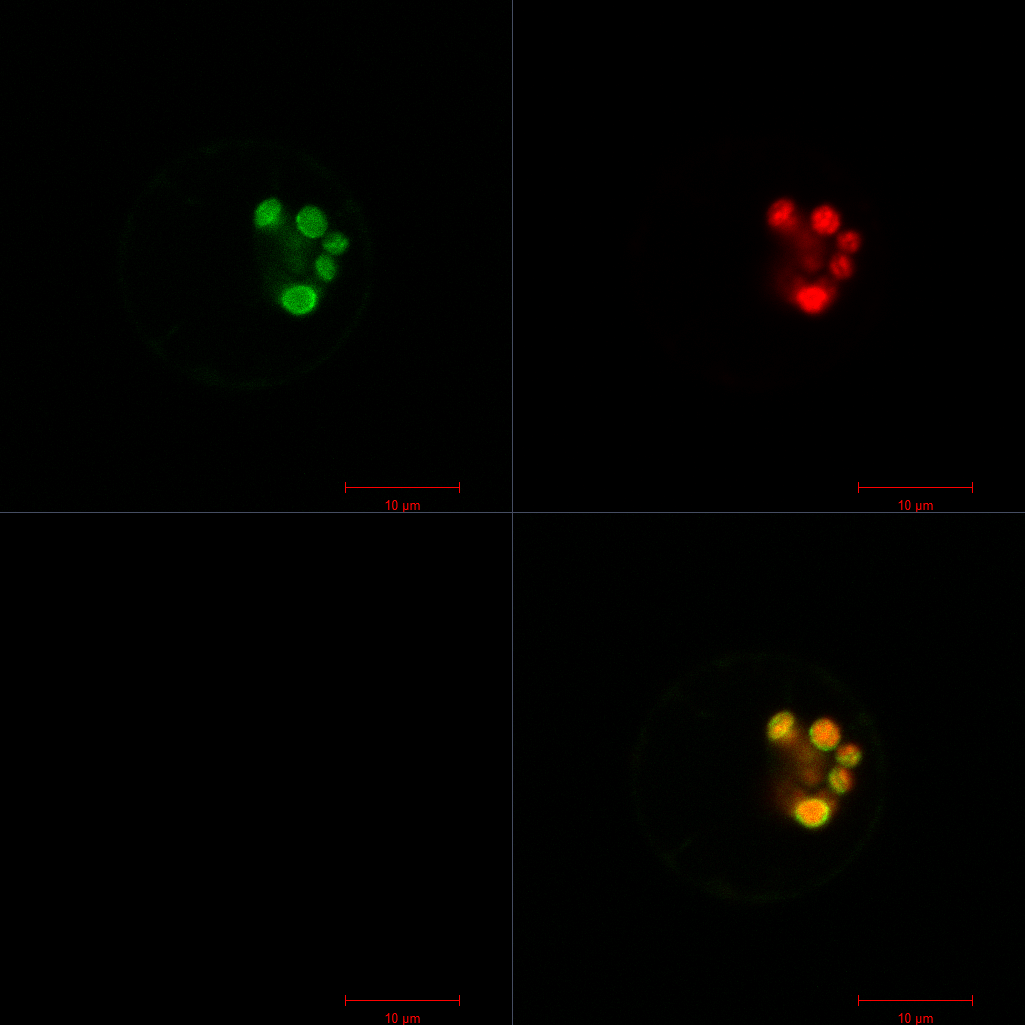

Supplement: Supplementary file 14 — Additional file 14. Original protoplast imaging files of barley. [file 12915_2022_1503_MOESM14_ESM.zip › Localization of protoplast in barley/HvHPT2_W5-8 max 10um.tif]

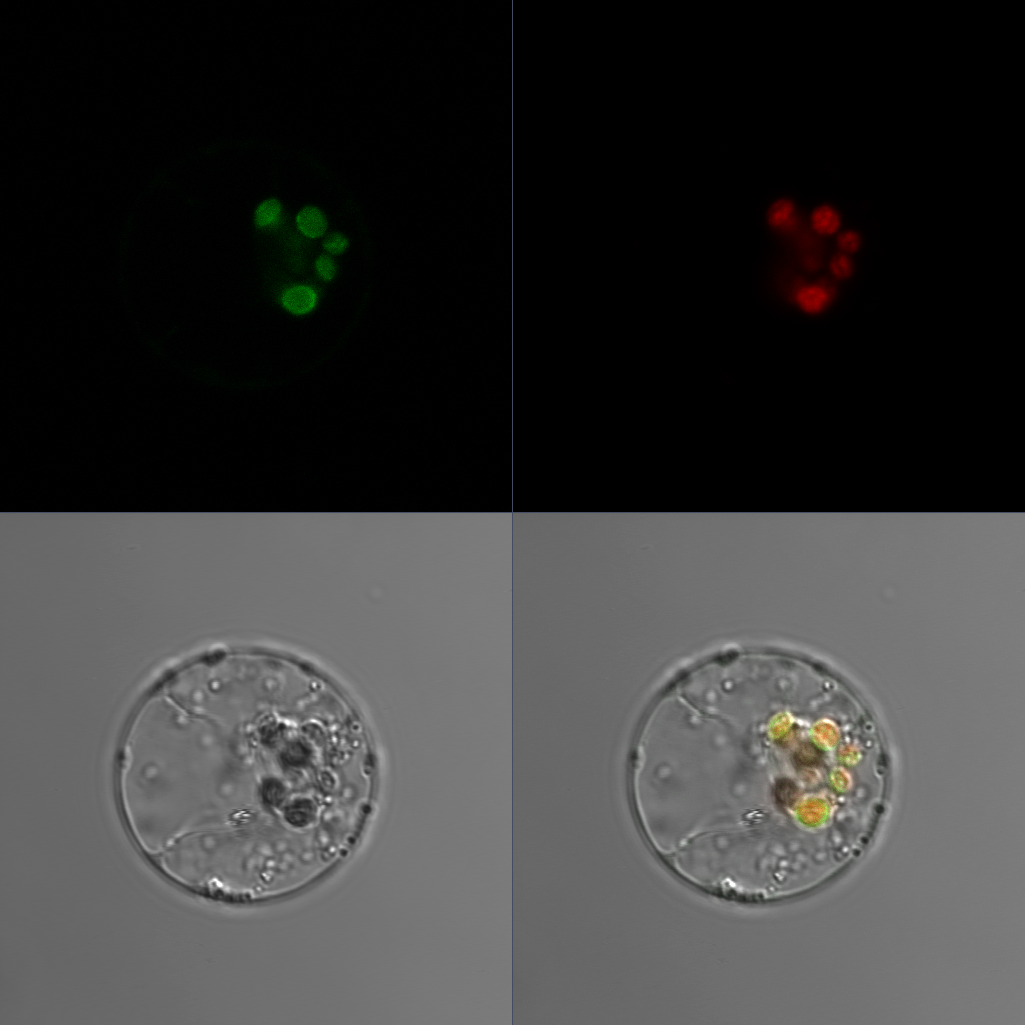

Supplement: Supplementary file 14 — Additional file 14. Original protoplast imaging files of barley. [file 12915_2022_1503_MOESM14_ESM.zip › Localization of protoplast in barley/HvHPT2_W5-8.tif]
